# Function Over Form: Modeling Groups of Inherited Neurological Conditions in Zebrafish

**DOI:** 10.3389/fnmol.2016.00055

**Published:** 2016-07-07

**Authors:** Robert A. Kozol, Alexander J. Abrams, David M. James, Elena Buglo, Qing Yan, Julia E. Dallman

**Affiliations:** ^1^Department of Biology, University of MiamiCoral Gables, FL, USA; ^2^Department of Human Genetics, John P. Hussman Institute for Human Genomics, Dr. John T. Macdonald Foundation, University of MiamiMiami, FL, USA

**Keywords:** zebrafish, disease modeling, autism spectrum disorder, intellectual disability, schizophrenia, ataxia, Charcot-Marie tooth, hereditary spastic paraplegia

## Abstract

Zebrafish are a unique cell to behavior model for studying the basic biology of human inherited neurological conditions. Conserved vertebrate genetics and optical transparency provide *in vivo* access to the developing nervous system as well as high-throughput approaches for drug screens. Here we review zebrafish modeling for two broad groups of inherited conditions that each share genetic and molecular pathways and overlap phenotypically: neurodevelopmental disorders such as Autism Spectrum Disorders (ASD), Intellectual Disability (ID) and Schizophrenia (SCZ), and neurodegenerative diseases, such as Cerebellar Ataxia (CATX), Hereditary Spastic Paraplegia (HSP) and Charcot-Marie Tooth Disease (CMT). We also conduct a small meta-analysis of zebrafish orthologs of high confidence neurodevelopmental disorder and neurodegenerative disease genes by looking at duplication rates and relative protein sizes. In the past zebrafish genetic models of these neurodevelopmental disorders and neurodegenerative diseases have provided insight into cellular, circuit and behavioral level mechanisms contributing to these conditions. Moving forward, advances in genetic manipulation, live imaging of neuronal activity and automated high-throughput molecular screening promise to help delineate the mechanistic relationships between different types of neurological conditions and accelerate discovery of therapeutic strategies.

## Introduction

As genetically tractable vertebrates (Streisinger et al., 1981) that share with humans many pathways targeted by FDA approved pharmaceuticals (Renier et al., 2007; Rihel et al., 2010) zebrafish are a powerful model for inherited neurological conditions, both in terms of delineating underlying mechanisms and developing therapeutic strategies. Their small size and optical transparency enable *in vivo* visualization of cell- and systems-level processes throughout early developmental stages (McLean and Fetcho, 2011; Rasmussen and Sagasti, 2016) while, precocious development of quantifiable behaviors (Brustein et al., 2003) and reduced complexity of the zebrafish nervous system (Goulding, 2009) simplify functional studies of neural circuits. These advantages combined with conserved vertebrate genetics lend themselves to keeping pace with the extraordinary discovery rate of genetic mutations that cause inherited neurological conditions in humans. With the sequencing of the human genome, the formation of worldwide consortia of human geneticists and clinicians and ever-cheaper sequencing technologies, these discoveries have revealed that many inherited disorders with related clinical diagnoses consist of large sets of rare molecular genetic variation (Buxbaum et al., 2012; Gonzalez et al., 2015). Here we focus on these parallel and synergistic frontiers of disease gene discovery and systems-level analyses in zebrafish that promise to yield insight into disease mechanisms and therapies.

To assess zebrafish as a model, we compare studies of two broad classes of inherited neurological conditions: developmental disorders and degenerative diseases, with each class presenting a spectrum of overlapping genotypes and phenotypes. For developmental disorders, we include Autism Spectrum Disorders (ASD), Intellectual Disability (ID) and Schizophrenia (SCZ) that can all affect executive functions, social and overall intellectual abilities (American Psychiatric Association, and DSM-5 Task Force, 2013). Such disorders can co-occur in the same individual (Amaral et al., 2011) supporting overlapping disease etiologies. The second general class we consider are a subset of degenerative diseases including Cerebellar Ataxia (CATX), Hereditary Spastic Paraplegia (HSP), spinal motor atrophy (SMA), amyotrophic lateral sclerosis (ALS) and Charcot-Marie Tooth Disease (CMT) that impair movement due to degeneration of long axon tracts (Züchner and Vance, 2005). In both developmental and degenerative cases there are examples of: (1) distinct clinical features within a given class that result from mutations in the same genes; and (2) shared clinical phenotypes produced by many different types of genetic mutation (Espinós and Palau, 2009; Kaufman et al., 2010; Timmerman et al., 2013; Vissers et al., 2016). For example, ASD affects roughly 1% of the population but even the most commonly mutated genes only account for 1–3% of ASD with hundreds of suspected causal loci (Miles, 2011; De Rubeis and Buxbaum, 2015; Geschwind and State, 2015). It is likely that these two groupings also have phenotypic overlap since age of onset and progression of symptoms varies within each grouping. For example, SCZ that we group with “developmental” disorders also shares symptoms with neurodegenerative conditions. None-the-less, by reviewing the literature on zebrafish models of these two groups of disorders, we hope to highlight the role we feel the zebrafish model has to play in revealing grouped mechanisms of shared clinical features that result from diverse genetic mutations.

## Comparing Human and Zebrafish Brains and Genetics

In addition to significant advantages of using zebrafish to model human disease, there are also challenges to modeling disease conditions in zebrafish. For example, in HSP symptoms are associated with degeneration of corticospinal tracts that have no clear homologous cell type in zebrafish. Moreover, for many human brain regions, the baseline studies to determine whether some of these brain regions function similarly to humans still need to be done. Finally, in terms of genetics, zebrafish have retained gene duplicates from a ray-finned fish whole genome duplication (Glasauer and Neuhauss, 2014) that likely provides both advantages (sub-functionalization of pleiotropic phenotypes) and disadvantages (genetic redundancy) for generating disease models.

### Conserved Brain Regions?

**Figure 1:**
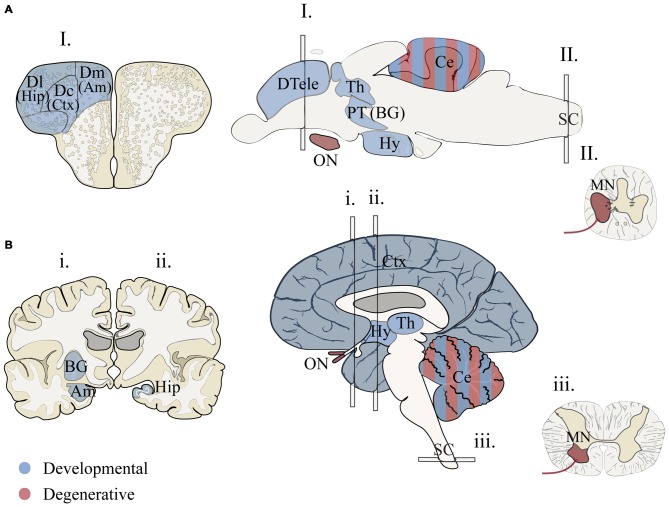
**Proposed structural homology between zebrafish and humans for brain regions associated with human neurodevelopmental disorders and neurodegenerative diseases. (A)** Adult zebrafish sections for I. telencephalon, brain and II. spinal chord. **(B)** Adult human sections for i., ii. telencephalon (anterior forebrain), brain and iii. spinal chord (transverse). Two hemi-sections were used (i. and ii.) to illustrate basal ganglia, hippocampus and amygdala. Regions associated with developmental disorders (blue) include cortical and subcortical structures that are vital for language, communication, memory, emotion and intellectual ability (Amaral et al., 2011; Bakhshi and Chance, 2015; Hampson and Blatt, 2015). Homologous forebrain regions for zebrafish are based on models that propose vertebrate structures that may be conserved for aspects of cognition and emotional behavior (Mueller and Wullimann, 2009; Mueller, 2012; Maximino et al., 2013; Filippi et al., 2014; Ganz et al., 2014; Wullimann, 2014). Conserved regions associated with axon degenerative diseases (red) include portions of the motor circuit and optic nerve (De Jonghe et al., 1997; Abrams et al., 2015). Zebrafish brain illustrations were adapted from Wullimann et al. (1996) and Mueller (2012). Am, amygdala; BG, basal ganglia; Ce, cerebellum; Ctx, cortex; Dc, dorsal central pallium; Dl, dorsal lateral pallium; Dm, dorsal medial pallium; DTele, dorsal telencephalon; Hip, hippocampus; Hy, hypothalamus; MN, motor neuron; PT, posterior tuberculum; Th, thalamus; ON, optic nerve.

Many brain regions relevant to human disease show molecular and structural homology in zebrafish (Figure 1). Gene expression patterns that molecularly define large-scale regions of fore-, mid-, hindbrain and spinal cord of the central nervous system (CNS) are generally conserved in vertebrates (Myers et al., 1986; Lumsden and Krumlauf, 1996; Wurst and Bally-Cuif, 2001; McLean et al., 2007; Wullimann et al., 2011). These major regions exhibit both neurochemical identities (e.g., neurotransmitters and receptors; Higashijima et al., 2004; Renier et al., 2007; Jones et al., 2015) and regional connectivity of sub-structures, including the thalamus (Mueller, 2012), optic tectum (Wullimann, 1994), hypothalamic regulatory nuclei (Herget et al., 2014), cerebellum (Hashimoto and Hibi, 2012), medulla oblongata (Kinkhabwala et al., 2011; Koyama et al., 2011), and spinal cord (Higashijima et al., 2004; Wen and Brehm, 2005). Determining homology between human and zebrafish forebrain structures is more challenging because of developmental differences affecting telencephalon topology (eversion vs. invagination) and the elaboration of the mammalian cerebrum (Striedter, 2005). Recent studies have made headway supporting the existence of zebrafish basal ganglia- (Filippi et al., 2014; Wullimann, 2014), cortex- (Ganz et al., 2014), amygdala- (Maximino et al., 2013) and hippocampus-like circuits (O’Connell and Hofmann, 2011; Maximino et al., 2013; Ganz et al., 2014). While these studies support the existence of structurally homologous brain regions, work is still needed to resolve connectivity and functional homology among fore- and midbrain structures. Furthermore some structures in the human brain appear absent in zebrafish including the pons (Wullimann et al., 2011), cortico-thalamic (Mueller, 2012) and cortico-spinal tracts (Babin et al., 2014). With these detailed analyses, we have the necessary anatomical map needed for neurological disease research that is quickly being enriched by functional studies to test the relevance to these brain regions for both zebrafish behaviors and human disorders.

To address the involvement of brain regions in a particular behavior, advances in functional imaging have been critical. Importantly, these studies focus on larval stages, 5–7 post fertilization, when it is possible to image the entire brain and the larva has already acquired an impressive repertoire of behaviors (Haesemeyer and Schier, 2015). For example, recent studies support functional homology of the zebrafish cerebellum during visual-motor behaviors (Hsieh et al., 2014; Matsui et al., 2014). These findings were made possible by combining behavioral experiments with both genetically encoded calcium imaging to visualize neuronal activity (Matsui et al., 2014) and loose patch recordings from cerebellar Purkinje neurons (Hsieh et al., 2014). These pioneering studies now open the door to sophisticated functional modeling of cerebellar-based neuropathies that can manifest in both neurodevelopmental disorders and neurodegenerative diseases, particularly CATX. Extending these functional technologies to forebrain circuits will be essential to support past studies suggesting similar cognitive and emotional functions exist in subdivisions of the zebrafish forebrain (Northcutt, 2006; O’Connell and Hofmann, 2011; Maximino et al., 2013).

### Conserved Genomes?

**Figure 2:**
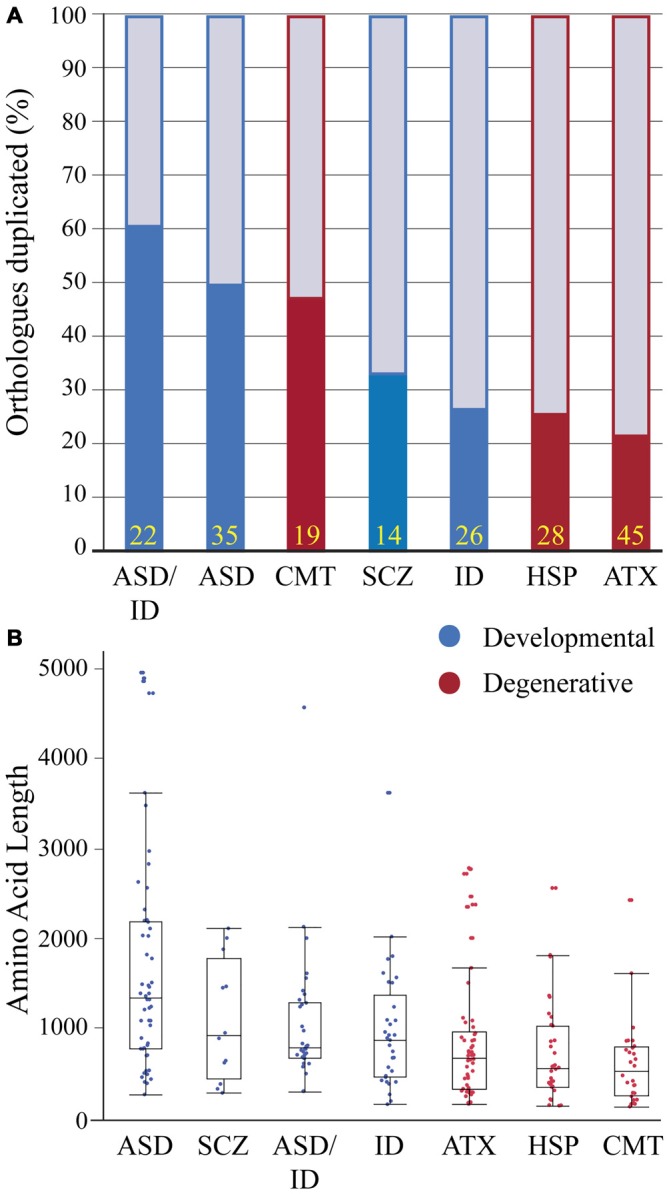
**Zebrafish orthologs of human neurological disease genes vary with respect to duplicate retention and average protein size. (A)** Gene duplicate retention rates in zebrafish are graphed for neurodevelopmental and neurodegenerative disease groups. Yellow numbers at the base of bars represent sample size. **(B)** Protein sizes of zebrafish orthologs of human disease genes with floating box plots (median with upper and lower quartile-box and range-whiskers). Note some larger proteins are outliers that fall outside of the calculated range. These gene sets for each human disease only incorporate a small percentage of associated genes and selection criteria varied because of the heterogeneity in genes linked to each disease and an emphasis on producing conservative lists. Each gene set was selected using data from research groups and review articles with the goal of including only the highest confidence disease genes based on either statistical thresholds and/or reoccurrence. Autism spectrum disorders (ASD) genes were chosen from the Simons Foundation Autism Initiative (SFARI.org) “high confidence” and “strong candidate” gene lists, which uses a multi-variable scoring analysis that includes sample size, statistical significance, replication, and functional analysis (Basu et al., 2009). ASD-intellectual disability (ID) genes were found in four separate reviews that provide evidence for reoccurrence in both ASD and ID (Kaufman et al., 2010; Krumm et al., 2014; Srivastava et al., 2014; Vissers et al., 2016). Schizophrenia (SCZ) genes were chosen from a SCZ genetics review, however this list is small and does not provide a confidence level for the disease contribution of each gene (Escudero and Johnstone, 2014). X-linked ID genes (Piton et al., 2013) and Charcot-Marie Tooth Disease (CMT; Timmerman et al., 2014) genes were chosen from recent meta-analyses to which we included a threshold of >5 cases per gene. Autosomal dominant and recessive and X-linked ataxia (ATX; Bird, 2016) and Hereditary Spastic Paraplegia (HSP; Fink, 2014) genes were chosen with well-known inheritance pedigrees. Human proteins for these gene lists were generated using BioMart (Kinsella et al., 2011) and the longest isoforms were used to identify zebrafish orthologs (Supplementary Material, Tables S1–7). Human proteins were then Blasted (Flicek et al., 2014) against the zebrafish proteome and ortholog information was recorded. Zebrafish proteins with low percent coverage, protein identity, e-value and ambiguous gene annotation (e.g., *gene-like, LOC1084*, etc.) were reciprocally blasted to confirm orthology.

Human and zebrafish genomes are highly conserved, with 76–82% of human disease genes present in zebrafish and an average of 20–24% of zebrafish genes duplicated (Howe et al., 2013). In some cases duplicates become sub-functionalized providing an advantage for studying pleiotropic phenotypes (Fleisch et al., 2008; Good et al., 2012; Lagman et al., 2015); in other cases, duplicates have redundant functions providing phenotypic buffering and complicating the generation of disease models (Hinits et al., 2012; Manoli and Driever, 2014). To determine if genes linked to a particular human neurological condition are enriched in gene duplicates we compared gene duplication rates in disease gene orthologs. Because past studies have shown duplicate enrichment in genes associated with neuronal development, signaling pathways and neuronal activity (Howe et al., 2013; Glasauer and Neuhauss, 2014), we hypothesized that neurodevelopmental duplicates would have a higher retention rate compared to neurodegenerative duplicates. Several gene sets showed a high duplicate retention frequency: over 60% (ASD-ID) and 45% (ASD and CMT; Figure 2A). In comparison, ID, ATX and HSP zebrafish orthologs had duplicate retention frequencies of 22–26% similar to the estimated genome-wide (20–24%) duplicate retention rate (Postlethwait et al., 2004; Howe et al., 2013). To determine if neurodevelopmental processes were enriched in sets with high duplicate retention rates we compared gene ortholog Gene Ontology (GO) term enrichments within orthologs sets (Mi et al., 2013). This program compares the frequency of a biological term (e.g., neuron development) with the expected frequency calculated for the genome. ASD (*n* = 35) and ASD-ID (*n* = 22) orthologs showed enrichments for GO terms associated with nervous system and neuronal development that confirm large studies analyzing extensive data sets of ASD and ID genes (Parikshak et al., 2013; Pinto et al., 2014). By contrast, CMT orthologs (*n* = 19) did not show any GO term enrichments overall, but those retained zebrafish CMT duplicates were enriched in genes associated with neuronal development (7/9 genes; Supplementary Material, Table S12). Therefore these genes may be associated with earlier onset cases of CMT, which can occur anywhere between birth and adulthood (De Jonghe et al., 1997). Although these gene sets are small and not statistically powerful, these rates show a general trend for retaining duplicates associated with neural development.

To continue comparing these ortholog classes we looked at the length of the longest protein-coding isoform for each disease gene ortholog. This parameter impacts modeling due to numerous exons and complex splicing. For example SH3 and multiple ankyrin repeat domain 3 (*SHANK3*) is a large gene and mouse *Shank3* knockout models exhibit variable phenotypes depending on whether a mutation is expressed in a predominant isoform (Peça et al., 2011; Zhou et al., 2016). Therefore we compared protein sizes between disease gene orthologs (Figure 2B). Between groups developmental disorders all had larger median protein sizes when compared to degenerative disease orthologs (Figure 2B). However, most notably proteins encoded by ASD genes were more than twice as long when compared to all degenerative disease proteins (Figure 2B). Because ASD genes have been suggested to encompass relatively large genes this result was perhaps not surprising (King et al., 2013; Uddin et al., 2014). Together these results on isoform length and duplicate enrichment suggests that ASD gene modeling in zebrafish will need to grapple with both extensive gene duplication and large complex genes that present challenges for knocking out all protein-coding isoforms and for generating rescue constructs.

## Neurodevelopmental and Neurodegenerative Zebrafish Models

### Developmental Disorders

Zebrafish models of developmental disorders have benefitted from the accessible embryonic stages and simplified nervous system that reveal an important role for signaling that patterns in the early embryo. Developmental disorders including ASD, ID and SCZ start to manifest phenotypically early in development and include deficits in social, learning and occupational functions (DSM-V). Because brain regions mediating human cognitive symptoms may lack parallels in zebrafish, modeling has focused on embryonic development and disorder comorbidities (such as sensory hypo-/hyper-sensitivity, sleep disruptions, and epilepsy) that still allow testing of etiological theories for these developmental disorders. These studies have paved the way for comprehensive functional assessments that link cellular- and circuit-level phenotypes to changes in behavior.

#### Molecular to Cellular Mechanisms: Signaling Pathways and Head Size

Mounting evidence links the etiology of neurodevelopmental disorders to embryonic stages. For example, teratogen exposure during gestation can cause developmental disorders (Arndt et al., 2005; Levy, 2011) and embryonic phenotypes in knockout mouse models provide support for an embryonic component underlying neurodevelopmental disorders (Knuesel et al., 2005; Lee et al., 2011; Durak et al., 2015). Zebrafish knockdown models of ASD and ID genes suggest that disrupted patterning of presumptive neural tissue in developmental disorders can occur as early as blastula stages (Yimlamai et al., 2005) and during gastrulation (De Rienzo et al., 2011; Turner et al., 2015). At the molecular level, these disruptions in patterning are likely due to changes in conserved signaling pathways. Several ASD and SCZ zebrafish models have investigated disease genes associated with the Wnt pathway (De Rienzo et al., 2011; Bernier et al., 2014; Brooks et al., 2014). For example, the Wnt interacting protein Chromodomain Helicase DNA binding domain 8 (CHD8) directly affects brain development during gastrulation and increases the size of the optic tectum, mirroring macroencephaly seen in ASD patients carrying *CHD8* mutations (Bernier et al., 2014; Sugathan et al., 2014). These genotype-specific features (e.g., macrocephaly) provide a phenotypic screen that can be used to investigate genetic classes within disorders. For example zebrafish *potassium channel tetramerization domain containing 13* (*kctd13*) was shown to have a dose-dependent affect in producing macrocephaly (knockdown) and microcephaly (overexpression) that supports a role for *KCTD13* copy number variants causing head size phenotypes (Golzio et al., 2012). Moreover, *Disrupted In Schizophrenia 1* (*DISC1)* interacts with canonical and non-canonical Wnt signaling and z*disc1* morphants and mutants exhibit disorganized axon tracts at larval stages that can be rescued by activating Wnt signaling (De Rienzo et al., 2011). These studies provide clear examples of utilizing zebrafish as an embryonic model to determine molecular and cellular mechanisms that define morphological phenotypes seen in individuals with developmental disorders.

#### Systems-Level Mechanisms: Disrupting the Balance of Neuronal Activity

Mechanisms affecting neuronal activity can contribute to neurodevelopmental disorders and zebrafish have been used to relate circuit-level changes in activity to behavior (Rubenstein and Merzenich, 2003; Eichler and Meier, 2008; Nelson and Valakh, 2015; Scharf et al., 2015). Circuit-level changes include disrupting the excitatory and inhibitory (E/I) balance, an operational set-point of excitation and inhibition within neural circuits that maintains functional behaviors (Borodinsky et al., 2004; Gatto and Broadie, 2010; Turrigiano, 2012; Vitureira et al., 2012; Davis, 2013). Zebrafish ASD and ID models have looked at E/I balance using transgenic fish lines expressing fluorescent glutamatergic (excitatory) and GABAergic (inhibitory) neurons (Kozol et al., 2015; Hoffman et al., 2016). Recently, Hoffman et al. found that populations of GABAnergic neurons were significantly decreased in *contactin associated protein-like 2* (*cntnap2ab*) mutants, recapitulating a mutant mouse *Cntnap2* model and suggesting that in the absence of cntnap2ab larvae fail to maintain inhibitory neuronal populations. This inhibitory decrease was shown to increase seizure susceptibility in *cntnap2ab^−/−^* mutants by applying a GABA receptor antagonist (Hoffman et al., 2016). In addition to seizure susceptibility, *cntnap2ab^−/−^* mutants had increased nighttime activity providing a circadian disruption for high-throughput drug screening. To identify potential therapies, they screened for drugs that reduced nighttime activity and identified a phytestrogen that restored wild type-like activity states. Like decreased inhibition, increased excitation is also known to contribute to developmental disorders. One well-studied example of this is augmented metabotropic glutamate receptor (mGluR) signaling in Fragile X Syndrome (Scharf et al., 2015). Similar to *Fragile x mental retardation 1* (*Fmr1*) knockout models in mice, a zebrafish *fmr1* knockdown model showed behavioral deficits that were ameliorated when treated with an mGluR inhibitor (Tucker et al., 2006). These studies demonstrate how zebrafish genetic models can be used to explore disorder etiology at multiple levels and efficiently test molecular theories for drug discovery.

#### Systems-Level Mechanisms: Comorbidities and Connecting Cells to Behavior

Individuals with developmental disorders are more likely to have accompanying medical conditions, or comorbidities, than typically developing individuals (Gurney et al., 2006; American Psychiatric Association, and DSM-5 Task Force, 2013; Chen et al., 2013a). Non-cognitive comorbidities such as sensory hypo- or hyper-sensitivity, epilepsy and gastrointestinal (GI) discomfort have revealed cellular-level mechanisms that may underlie behavioral phenotypes in developmental disorders. Several zebrafish knockdowns models of ASD, ID and epilepsy genes have looked at impaired touch sensitivity. Knockdown models of the ASD genes *autism susceptibility candidate 2* (*auts2*) and *shank3a* exhibit hyposensitivity with concomitant neuronal cell death and morphological changes in skin innervating sensory neurons (Oksenberg et al., 2013; Kozol et al., 2015). Also exploring sensitivity, *chromodomain helicase DNA binding protein 2* (*chd2*) knockdowns and *sodium channel, voltage gated, type II, alpha* (*scn1lab*) knockouts display hyper-excitable phenotypes that are characterized by extended or disorganized swimming with epileptiform-like activity in the brain (Baraban et al., 2013; Suls et al., 2013; Galizia et al., 2015). These epileptic swimming bouts provide a stereotyped behavior for high-throughput drug screening. Such a screen in *scn1lab* mutants identified anti-histamine clemizole as a novel anti-epileptic drug (Baraban et al., 2013). Although more focus has been paid to conditions such as epilepsy, other comorbidities like GI distress in ASD have yet to be investigated comprehensively (Hsiao, 2014; Bresnahan et al., 2015). For instance, *chd8* morphants have a decrease in HuC/D positive enteric neurons innervating the gut and have impaired gut motility (Bernier et al., 2014). Again this example provided a cellular to systems level mechanism for GI distress seen in a majority of ASD patients carrying a *CHD8* mutation. These examples all show the utility of zebrafish for studying comorbidities that impact the quality of life of large cohorts of patients; therefore a better understanding of the basis for these comorbidities would likely improve patient care.

### Hereditary Neurodegenerative Disorders

Some common cellular mechanisms underlying degenerative diseases have been elucidated through gene discovery and zebrafish modeling of rare hereditary diseases. The degeneration of axon tracts in the central and peripheral nervous system are a clinical feature in neurodegenerative disorders such as Charcot-Marie-Tooth disease type 2 (CMT2), HSP, SMA or spinal muscle atrophy (SMA), ALS, as well as some forms of CATX which have phenotypic and mechanistic overlap (Züchner and Vance, 2005; Timmerman et al., 2013; Bargiela et al., 2015; Burté et al., 2015). The early development of zebrafish peripheral, motor and sensory neurons provide a foundation that has been used to dissect molecular mechanisms at both the cellular and systems level especially in models of SMA and ALS (McGown et al., 2013; Wiley et al., 2014). Using an innovative strategy to develop SMA therapies, one study used a high-throughput synthetic genetic array (SGA) screen in fission yeast to identify gene networks that when targeted with drugs reversed motor axon outgrowth deficits in a zebrafish SMA model (Wiley et al., 2014). Zebrafish models of these neurodegenerative diseases have also focused on molecular mechanisms such as axonal transport, mitochondrial dynamics, and autophagy, while also measuring morphological changes at the systems level such as alterations at the neuromuscular junction, degeneration of motor and sensory neurons, and disruptions of Purkinje cell (PC) development.

#### Cellular Level Mechanisms: Axonal Transport

A subset of the causative genes in these disorders are directly involved in axonal transport processes (Timmerman et al., 2013). The optical transparency of zebrafish and transgenic lines available make zebrafish an ideal model to study the relationship between axonal transport and axon degeneration *in vivo* (Plucińska et al., 2012; O’Donnell et al., 2014). Mutations in *Kinesin Family member 5A* (*KIF5A*), a molecular motor for transporting microtubule-mediated cargo, have been reported in both CMT2 (Crimella et al., 2012), and HSP patients (Reid et al., 2002; Fichera et al., 2004). A* kif5a* mutant zebrafish shows decreased touch response, and defective sensory neuronal maintenance all within the larval stages of development (Campbell et al., 2014). Furthermore the authors found that kif5a specifically affects the transport and distribution of mitochondria in neurons, but not lysosomes or presynaptic vesicles. Dominant mutations in *Atlastin GTPase 1* (*ATL1*) encoding atlastin-1 cause an early onset form of HSP (Dürr et al., 2004). Morpholino knockdown of *atl1* in zebrafish causes decreased mobility in larval fish and specifically disrupts axon tracts of spinal motor neurons (Fassier et al., 2010). Fassier et al. (2010) further demonstrated that the phenotype is the result of altered BMP signaling and that atlastin may play a role in BMP receptor trafficking. This link to BMP receptor trafficking suggested blocking BMP receptors as a therapeutic strategy that indeed ameliorated both cellular and behavioral phenotypes in the *atl1* morphant zebrafish model. These zebrafish models support axonal transport as a cellular mechanism that could explain why long axons in CMT2 and HSP are primarily affected by genetic mutations in genes associated with transport processes.

#### Cellular Level Mechanisms: Mitochondrial Neuropathies

Mitochondrial dysfunction is another common mechanism in neurodegeneration. Dominant mutations in *Mitofusin 2* (*MFN2*) are the primary cause of axonal degeneration in Charcot-Marie-Tooth Neuropathy (CMT2), and MFN2 has been implicated in the fusion and transport of mitochondria in neurons (Chen et al., 2003; Züchner et al., 2004; Baloh et al., 2007). Murine *Mfn2* knockout and knock-in models are embryonic or postnatal lethal and do not develop a peripheral neuropathy, however a conditional knockout model did produce cerebellar degeneration and neonatal lethality (Chen et al., 2003, 2007; Strickland et al., 2014). In contrast, a stable mfn2^L285X^ loss-of-function zebrafish model does recapitulate the motor neuron degenerative phenotype showing progressive loss of swimming ability, loss of neuromuscular junctions (NMJs), and early lethality by 1 year of age (Chapman et al., 2013). The authors found that the transport of mitochondria is disrupted in cultured motor neurons from the homozygous mfn2^L285X^ at 24 hpf suggesting that a primary transport defect occurs before the onset of symptoms. In addition to axon degeneration of motor neurons a portion of patients with* MFN2* mutations also develop optic atrophy (Züchner et al., 2006). The optic nerve seems to be particularly susceptible to mitochondrial dysfunction and is often affected in clinical spectrum phenotypes classified as mitochondrial optic neuropathies (Yu-Wai-Man et al., 2009). Recessive mutations in the nuclear encoded mitochondrial gene *Optic Atrophy 3* (*OPA3*) cause a spectrum disorder classified as Costeff syndrome and includes optic atrophy, ataxia, extra pyramidal dysfunction, and increased urinary excretion of 3-methylglutaconic acid (MGC; Costeff et al., 1989). Zebrafish *opa3* null mutants show increased MGC at both 5 dpf and at 2–5 months (Pei et al., 2010). At 1 year they show decreased optic nerve thickness and retinal ganglion cell density. Mutants have detectable changes in movement behaviors at larval stages and adults show loss of horizontal swimming. The authors speculate that the swimming phenotype can be attributed to ataxia, however TUNEL and histological staining of the cerebellum did not reveal any abnormalities. A third disease gene linked to mitochondrial dynamics and a spectrum of degenerative neurological conditions that include optic atrophy, CMT and cerebellar degeneration is *Solute Carrier family 25, member 46 SLC25A46*; Abrams et al., 2015). Studies in zebrafish and patient stem cells linked disruption of *slc25a46* to reduced mitochondrial fission, altered distribution of mitochondria in motor neurons, and defective maintenance of neuronal processes. Even though cellular phenotypes were dramatic, swimming deficits in *slc25a46* morphants were mild.

#### Cellular Level Mechanisms: Cerebellar Purkinje Neurons

Ataxia and associated sensory and motor phenotypes result from genetic mutations that affect various cell types within the spinocerebellar circuit. Cerebellar PCs are commonly affected and appear especially sensitive to peroxisomal dysfunction associated with PC loss or cerebellar atrophy (Akbar and Ashizawa, 2015). Consistent with this, zebrafish ataxia models, such as *sorting nexin 14* (*snx14*) morphants show decreases in PC progenitors while *cwf19-like 1* (*cwf1911*) morphants show disruptions in overall hindbrain morphology (Burns et al., 2014; Akizu et al., 2015). Alternatively, one group has focused on primary motor neurons for functional studies of *Potassium Channel, voltage gated shaw related subfamily c, member 3* (*KCN3*) mutations that cause Spinocerebellar ataxia type 13 (Waters et al., 2006). They found that zebrafish *kcn3a* is expressed in the primary motor neurons and overexpression of a dominant negative version of this potassium channel decreases in neuronal excitability during fictive swimming (Issa et al., 2012). To follow up this study, Issa et al. also investigated the affect of *KCN3* mutations associated with infant onset ataxia. Overexpression of two human *KCN3* mutations demonstrated axonal defects that were only found in a mutation associated with infantile onset of SCA13. Given that neurodegenerative phenotypes become more severe with age, there has been doubt as to whether modeling these diseases in zebrafish larvae would be informative. The studies reviewed above indicate that indeed it is possible to gain functional insight into the basic biology of these disease genes from modeling in the larva. As a newer model, however, approaches in zebrafish are not quite as standardized as those in longer established models like *Drosophila* and mouse resulting in the diversity of modeling strategies employed by different researchers to model these inherited neurological conditions.

## Lessons Learned From Modeling Inheritable Disease Genes

### Homeostatic Plasticity: Unlinking Cellular and Systems Level Phenotypes

In some less common examples, genetic mutations cause cellular- and molecular-level phenotypes without leading to behavioral phenotypes. In zebrafish *where’s waldo* and *strumpy* mutants, neuromuscular synaptogenesis is defected but the mutants exhibit normal motility (Hutson and Chien, 2002; Panzer et al., 2005). Similar phenomena have been observed in mouse hypoxanthine-guanine phosporibosyltransferase (HPRT) and HPRT-adenine phosporibosyltransferase (APRT) mutant models of Lesch-Nyhan syndrome genes that produced an expected drop in dopamine but lacked self mutilation behaviors (Kuehn et al., 1987; Engle et al., 1996; Jinnah et al., 1999). These observations indicate that molecular- and cellular-level phenotype does not always correspond to behavioral phenotype, suggesting the existence of compensatory mechanisms at the systems-level. Besides genetic compensation from other genes, the nervous system also has remarkable capacity to stabilize its functions via homeostatic plasticity. Compensatory homeostatic plasticity operates on multiple levels, regulating synaptogenesis, synaptic strength and intrinsic excitability to stabilize neural circuit output in the context of genetic and/or environmental perturbations (Turrigiano, 2012; Vitureira et al., 2012; Davis, 2013). In zebrafish *strumpy^p37er^* mutants have enlarged NMJ acetylcholine receptor clusters are compared to wild-type (Panzer et al., 2005), but lack motor phenotypes, indicating homeostatic plasticity. In addition, defective homeostatic plasticity has been associated to a variety of human neurological diseases, including ASD, ID and Fragile X Syndrome (Rubenstein and Merzenich, 2003; Eichler and Meier, 2008; Gatto and Broadie, 2010; Yizhar et al., 2011; Wondolowski and Dickman, 2013; Nelson and Valakh, 2015). Mutant animal models with cellular but not behavioral phenotype have the potential to shed light on mechanisms of homeostatic plasticity at the systems-level. Given the diversity of molecular genetic pathways that contribute to developmental and degenerative disorders, a potential therapeutic target is to boost compensatory mechanisms that act to re-establish systems-level function.

### Genetic Buffering: Molecular Compensation for Genetic Lesions

It is also common, though not necessarily well-represented in the literature, for knockout models to lack phenotypes. In a zebrafish study that generated mutant lines with 32 distinct lesions in 24 genes, most of the mutants exhibit a wild-type phenotype (Kok et al., 2015). Rather than a unique characteristic of zebrafish, such phenotypic buffering is found across single-celled to multi-cellular organisms. For example, >70% in fission yeast and >80% in bakers yeast, of genes can be individually mutated with little effect on haploid viability in the laboratory setting (Kim et al., 2010)^1^. Also in mice, though it is hard to accurately estimate the proportion of knockout mice without detectable phenotypes due to a lack of publications, the number is considerable (Barbaric et al., 2007). This phenomenon can be explained by the recent finding that other genes in a regulatory network can provide genetic compensation in mutants (Rossi et al., 2015). Such compensation can vary depending on genetic background (Gerlai, 1996; Pearson, 2002). Another recent study in zebrafish shows that the oncogenic *B-RAF* proto-oncogene (*BRAF^V600E^*) mutations rarely covert carrier cells into cancer cells unless in a *p53* mutant background (Kaufman et al., 2016). The importance of genetic background for gene regulatory compensation likely contributes to disease penetrance and expressivity in humans as well (Zlotogora, 2003; Andersen and Al-Chalabi, 2011; Cooper et al., 2013; Persico and Napolioni, 2013), and suggests the importance to move from analyzing single gene to systems-level analysis of gene regulatory networks in disease models (Döhr et al., 2005; Barabási et al., 2011; Chen et al., 2014; Wiley et al., 2014; Marbach et al., 2016).

### Broad Gene Expression; Local Pathology

**Figure 3:**
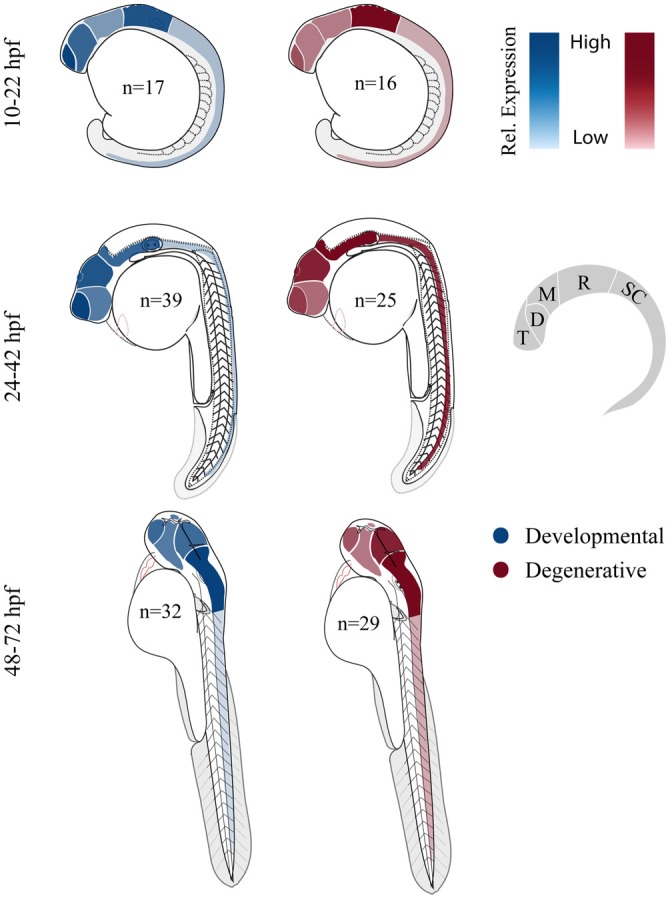
**Comparing relative central nervous system (CNS) gene expression for developmental and degenerative disease gene orthologs in zebrafish.** Previously published *in situ* hybridization data was collected from the zebrafish model organism database (ZFIN.org). Expression level was scored for each gene proportionally from 0 to 3; 0, no expression; 1, expression; 2, moderate expression; and 3, high level of expression, (i.e., gene *x* showed expression in the telencephalon (1) at 12 hpf and comparatively high expression in the telencephalon (3) at 36 hpf). Zebrafish larvae illustrations were adapted from Kimmel et al. (1995). D, diencephalon; M, mesencephalon; R, rhombencephalon; T, telencephalon and SC, spinal cord (De Jonghe et al., 1997; Kudoh et al., 2001; Thisse et al., 2001; Wurst and Bally-Cuif, 2001; Groth et al., 2002; Rauch et al., 2003; Thisse and Thisse, 2004, 2005; Croushore et al., 2005; Imamura and Kishi, 2005; Meyer et al., 2005; Thompson et al., 2005; Yimlamai et al., 2005; Liu et al., 2006; Mendelsohn et al., 2006; Meyer and Smith, 2006; Cheng et al., 2007; George et al., 2007; Goruppi et al., 2007; Katsuyama et al., 2007; Patten et al., 2007; Anichtchik et al., 2008; Stuebe et al., 2008; Sun et al., 2008; Yoshida and Mishina, 2008; Zhou et al., 2008; Emond et al., 2009; Ferrante et al., 2009; Monnich et al., 2009; Patten and Ali, 2009; Titus et al., 2009; Wood et al., 2009; Appelbaum et al., 2010; Davey et al., 2010; Fassier et al., 2010; Rissone et al., 2010; Takada and Appel, 2010; Mapp et al., 2011; Yeh et al., 2011; Artuso et al., 2012; Dresner et al., 2012; Gomez et al., 2012; Imai et al., 2012; Mueller, 2012; Pujol-Martí et al., 2012; Xing et al., 2012; Yanicostas et al., 2012; Baraban et al., 2013; Campbell and Marlow, 2013; Haug et al., 2013; Ng et al., 2013; Recher et al., 2013; Suls et al., 2013; Vatine et al., 2013; Bernier et al., 2014; Garbarino et al., 2014; Housley et al., 2014; Hsieh et al., 2014; Galizia et al., 2015; Kozol et al., 2015; Wakayama et al., 2015).

Both developmental and degenerative diseases are associated with dysfunction in specific regions of the CNS (Figure 1). None-the-less most genes have heterogeneous spatial and temporal expression patterns that extend well beyond the windows of time and specific neural circuits associated with the disorder. Therefore we did a limited meta-analysis of mRNA expression of zebrafish disease orthologs. Unfortunately a zebrafish gene expression atlas does not exist and the most complete data sets only span embryonic development. Therefore we chose to compile previously published *in situ* hybridization data and determine the relative enrichment of gene expression in brain regions and spinal cord (Figure 3; Suplementary Material, Tables S13–18). During the first day of development, both gene sets show enriched expression in the hindbrain with the developmental set having enriched expression in the forebrain. By the second and third day, expression patterns become broader showing similar enrichment throughout the brain at these stages of morphogenesis and circuit formation. Broad CNS expression patterns suggest that genes play functional roles throughout development and across the nervous system despite being associated with symptoms that disrupt specific circuits during particular times of life.

## Looking Forward: Neural Circuits, Behavior, and Therapy

Because of the large number of rare mutations linked to inherited nervous system diseases, an important frontier for disease modeling is strategies that leverage to make stable F_0_ mutant models of inherited neurological disorders (Jao et al., 2013; Shah et al., 2015). Such models enable the rapid screening of candidate disease genes for whether they produce disease relevant phenotypes in the zebrafish model. To this end, many groups currently augment their MO studies by demonstrating similar phenotypes in F_0_ mutants (Bernier et al., 2014; Aspatwar et al., 2015; Bögershausen et al., 2015; O’Rawe et al., 2015; Wheeler et al., 2015; Xing et al., 2015). Still others have found mismatches between morphant and stable mutant phenotypes (Kok et al., 2015; Rossi et al., 2015; Stainier et al., 2015). Clearly in some cases these mismatches ascribed to non-specific effects of the morpholino (Kok et al., 2015) can also be explained by compensatory mechanisms masking the phenotype in the stable mutant (Rossi et al., 2015). To address the challenges of gene duplicates and multiple mutation causes of disease (Shah et al., 2015), several labs have further pioneered a strategy to pool guides targeting multiple genes and inject them together to efficiently screen multiple mutations in the F_0_ generation. Combined with a large repertoire of behaviors that develop within 5 days of fertilization and diverse transgenic lines for rapid screening of cellular phenotypes, F_0_ CRISPR zebrafish mutagenesis promises to contribute significantly to our understanding of genetic variation linked to nervous system disorders.

To model specific patient missense mutations and to better understand the basic biology of disease genes by tagging them *in vivo* or create conditional mutant alleles, several groups have also recently pioneered the use CRISPR/Cas9 for more sophisticated genome engineering. One novel strategy effectively “enhancer traps” a gene of interest by replacing the last exon with an engineered last exon encoding the C-terminal end of the coding sequence in frame with a cleavable p2A sequence followed by a fluorescent reporter (Li et al., 2015). In this way, the spatial and temporal expression dynamics of the protein can be captured. Also recently, Hoshijima et al. (2016) have developed streamlined strategies to precisely edit the genome and generate conditional mutant alleles flanked by LoxP sites. Such conditional mutant alleles have been used to great effect in mouse models to test when the mutation acts to produce different disease phenotypes. For example, in a mouse *Shank3* autism model, rescuing the mutant Shank3 protein in the adult was sufficient to rescue social interactions and excessive grooming but not anxiety and repetitive motor behaviors (Mei et al., 2016).

The development of approaches that enable monitoring of behavior-relevant neural circuits in the intact larvae will be a boon for modeling inherited neurological disease (Ahrens et al., 2013; Fosque et al., 2015; Randlett et al., 2015; Dunn et al., 2016). Such approaches have the significant advantage of being unbiased. While many of these systems-level analyses use light-sheet or high-end microscopy to capture data (Ahrens et al., 2013; Fosque et al., 2015; Dunn et al., 2016), others use standard confocal microscopy to identify relevant brain circuits (Randlett et al., 2015) that are often spatially distributed across the nervous system. Several groups have also made concerted efforts towards establishing brain atlases to structurally and functionally annotate the brain (Ronneberger et al., 2012; Turner et al., 2014; Randlett et al., 2015) which is crucial since the ability to interpret imaging data is only as good as our understanding of brain regions (Arrenberg and Driever, 2013). Once these circuits are identified, they can then be studied in greater depth using *in vivo* calcium imaging with genetically encoded calcium indicators- (GECIs; Chen et al., 2013b), laser or enzymatic ablation of parts of the circuit (Liu and Fetcho, 1999; Tabor et al., 2014; Chen et al., 2016), electrophysiological recordings (Koyama et al., 2011; Baraban, 2013; Johnston et al., 2013; Wen et al., 2013), and optogenetics (Wyart et al., 2009; Kimura et al., 2013) to dissect circuit properties. Most of these approaches have yet to be broadly applied to zebrafish models of disease but once applied more broadly they promise to significantly contribute to our understanding of systems-level neural circuit mechanisms that contribute to symptoms of inherited neurological disorders.

In addition to genetic screens, due to their small size and their tendency to absorb drugs added directly to the water, zebrafish larvae are uniquely amenable for high-throughput drug screens (Rihel et al., 2010; Rihel and Schier, 2013; Bruni et al., 2014). High-throughput behavioral screens in zebrafish have enabled the classification of neuro-active drugs with respect to their impact on whole organism behavior. The ability to screen compounds in this manner is crucial since neuroactive drug discovery is still more empirical—a matter of what works—rather than rational—a matter of what makes sense based on chemistry and known molecular targets (Bruni et al., 2014). As highlighted in neurodevelopmental and neurodegenerative sections above, several disease models have made great use of the ability to use drugs to enhance or suppress mutant phenotypes as a means to identify therapeutic strategies (Fassier et al., 2010; Baraban et al., 2013; Hoffman et al., 2016).

## Conclusion

The continued expertise and innovations of zebrafish genetic and developmental tools will continue to make zebrafish an attractive neurological disease model. Going forward, combining standard assays that allow comparisons across models with newer approaches would be ideal to enable a better understanding of the molecular, cellular, and systems-level groupings of these neurological conditions. Finally, zebrafish will certainly contribute to consortia of research groups that use multiple animal models for discovering essential molecular to circuit level mechanisms underlying neurological disease.

## Author Contributions

RAK conducted all meta-analyses, made all figures and wrote introductory and developmental disorder sections and conducted extensive edits to coordinate sections. AJA wrote the bulk of the neurodegenerative section with the exception of the ataxia section that was written by EB. DMJ wrote comorbidity section that centered on GI distress in developmental disorders. QY wrote the lessons learned section. JED conceived the scope of the review, wrote frontiers section and helped RAK to conduct extensive edits to coordinate sections.

## Funding

This work was supported by support from the National Institutes of Health Institute of Mental Health R03MH103857 to JED and from the Institute of General Medicine, an IMSD graduate fellowship from parent grant R25GM076419 to DMJ.

## Conflict of Interest Statement

The authors declare that the research was conducted in the absence of any commercial or financial relationships that could be construed as a potential conflict of interest.

## Footnotes

^1^http://www.yeastgenome.org
