# Supplementary material for: Function Over Form: Modeling Groups of Inherited Neurological Conditions in Zebrafish
Source: Front Mol Neurosci. 2016 Jul 7;9:55. doi: 10.3389/fnmol.2016.00055 (PMC4935692; doi:10.3389/fnmol.2016.00055)
Supplement: Supplementary file 1 [file DataSheet_1.docx]

**SUPLEMENTAL DATA**

**Table 1. Autism Spectrum Disorder Gene Orthologues Blast results. Ch., chromosome; aa, protein length in amino acids; Iso., isoforms (ENSMBL.org/NCBI.gov).**

| **Gene** | **Ch.** | **aa** | **Query Cover %** | **% Protein ID** | **Iso.** | **E-value** | **Zebrafish Protein NCBI/ENSEMBL ID** | **Human Protein NCBI/ENSEMBL ID** |
| --- | --- | --- | --- | --- | --- | --- | --- | --- |
| ank2a | 1 | 4847 | - | 70 | 1 | - | [ENSDART00000021693](http://useast.ensembl.org/Danio_rerio/Transcript/Summary?db=core;g=ENSDARG00000009026;r=1:13386954-13473738;t=ENSDART00000021693;tl=xtVxQvMUCCQxUzev-1344268-292587305) | ENSP00000349588 |
| ank2b | 7 | 4714 | 72 | 72 | 11 | 0 | XP_009305106.1 | ENSP00000349588 |
| ankrd11 | 7 | 2819 | 96 | 51 | 2 | 0 | [XP_005166635.1](http://www.ncbi.nlm.nih.gov/protein/528486070?report=genbank&log$=prottop&blast_rank=1&RID=73D8JR6X01R) | ENSP00000301030 |
| arx | 24 | 453 | 94 | 78 | 1 | 0 | [NP_571459.1](http://www.ncbi.nlm.nih.gov/protein/18858285?report=genbank&log$=prottop&blast_rank=1&RID=FN9G6W2401R) | ENSP00000368332 |
| arxl | 21 | 385 | 89 | 54 | 1 | 0 | [XP_002667096.1](http://www.ncbi.nlm.nih.gov/protein/292628755?report=genbank&log$=prottop&blast_rank=2&RID=FN9G6W2401R) | ENSP00000368332 |
| ashl1 | 19 | 2962 | 76 | 67 | 3 | 0 | [XP_692254.5](http://www.ncbi.nlm.nih.gov/protein/528509336?report=genbank&log$=prottop&blast_rank=1&RID=75ZKZFYH01R) | ENSP00000357330 |
| bcl11aa | 13 | 829 | 99 | 75 | 2 | 0 | [NP_001035481.1](http://www.ncbi.nlm.nih.gov/protein/94536705?report=genbank&log$=prottop&blast_rank=1&RID=73D8JR6X01R) | ENSP00000338774 |
| bcl11ab | 6 | 776 | 99 | 62 | 1 | 0 | [NP_001094421.1](http://www.ncbi.nlm.nih.gov/protein/155369325?report=genbank&log$=prottop&blast_rank=2&RID=73D8JR6X01R) | ENSP00000338774 |
| cacna1ha | 3 | 2190 | 92 | 61 | 2 | 0 | [XP_009297960.1](http://www.ncbi.nlm.nih.gov/protein/688544239?report=genbank&log$=prottop&blast_rank=7&RID=73D8JR6X01R) | ENSP00000334198 |
| cacna1hb | 1 | 2197 | 92 | 58 | 6 | 0 | [XP_009304662.1](http://www.ncbi.nlm.nih.gov/protein/688531564?report=genbank&log$=prottop&blast_rank=10&RID=73D8JR6X01R) | ENSP00000334198 |
| cacna2d3-1 | 11 | 1082 | 98 | 76 | 3 | 0 | [XP_002663860.](http://www.ncbi.nlm.nih.gov/protein/326671946?report=genbank&log$=prottop&blast_rank=1&RID=73D8JR6X01R) | ENSP00000288197 |
| cacna2d3-2 | 8 | 1080 | 97 | 77 | 3 | 0 | [XP_009302617.1](http://www.ncbi.nlm.nih.gov/protein/688569631?report=genbank&log$=prottop&blast_rank=2&RID=73D8JR6X01R) | ENSP00000288197 |
| cdkl5 | 11 | 1080 | 99 | 63 | 3 | 0 | [NP_001139240.1](http://www.ncbi.nlm.nih.gov/protein/224809224?report=genbank&log$=prottop&blast_rank=1&RID=FN9G6W2401R) | ENSP00000485184 |
| chd2 | 18 | 1813 | 95 | 71 | 4 | 0 | [XP_009291898.1](http://www.ncbi.nlm.nih.gov/protein/688597113?report=genbank&log$=prottop&blast_rank=1&RID=73D8JR6X01R) | ENSP00000377747 |
| chd8 | 2 | 2554 | 94 | 62 | 3 | 0 | [XP_005171370.1](http://www.ncbi.nlm.nih.gov/protein/528471505?report=genbank&log$=prottop&blast_rank=1&RID=75ZKZFYH01R) | ENSP00000451601 |
| cntn4 | 6 | 1028 | 99 | 64 | 2 | 0 | [XP_009300931.1](http://www.ncbi.nlm.nih.gov/protein/688561681?report=genbank&log$=prottop&blast_rank=1&RID=73D8JR6X01R) | ENSP00000380602 |
| cntnap2a | 24 | 1316 | 99 | 71 | 1 | 0 | [NP_001268920.1](http://www.ncbi.nlm.nih.gov/protein/530354704?report=genbank&log$=prottop&blast_rank=1&RID=73D8JR6X01R) | ENSP00000354778 |
| cntnap2b | 2 | 1315 | 99 | 65 | 1 | 0 | [NP_001289171.1](http://www.ncbi.nlm.nih.gov/protein/694871376?report=genbank&log$=prottop&blast_rank=3&RID=73D8JR6X01R) | ENSP00000354778 |
| ctnnd2a | 24 | 1221 | 98 | 77 | 2 | 0 | [XP_002666700.2](http://www.ncbi.nlm.nih.gov/protein/528519872?report=genbank&log$=prottop&blast_rank=6&RID=73D8JR6X01R) | ENSP00000307134 |
| ctnnd2b | 2 | 1203 | 98 | 77 | 6 | 0 | [XP_009296848.1](http://www.ncbi.nlm.nih.gov/protein/688537279?report=genbank&log$=prottop&blast_rank=1&RID=73D8JR6X01R) | ENSP00000307134 |
| cul3a | 2 | 768 | - | 97 | 1 | 0 | [ENSDARP00000056881](http://useast.ensembl.org/Danio_rerio/Transcript/ProteinSummary?db=core;t=ENSDARP00000056881;tl=MRK4uhARfuCpAK0B-1341885-292187097) | ENSP00000264414 |
| cul3b | 15 | 768 | 99 | 98 | 2 | 0 | NP_001177414.1 | ENSP00000264414 |
| deaf1 | 25 | 528 | 99 | 53 | 2 | 0 | [NP_001124251.1](http://www.ncbi.nlm.nih.gov/protein/195546790?report=genbank&log$=prottop&blast_rank=1&RID=73D8JR6X01R) | ENSP00000371846 |
| dmd | 1 | 3609 | 99 | 58 | 10 | 0 | [XP_009304312.1](http://www.ncbi.nlm.nih.gov/protein/688531359?report=genbank&log$=prottop&blast_rank=1&RID=FN9G6W2401R) | ENSP00000354923 |
| dscama | 10 | 2025 | 78 | 99 | 2 | 0 |  | ENSP00000383303 |
| dscamb | 15 | 2020 | 99 | 78 | 2 | 0 | [XP_009289749.1](http://www.ncbi.nlm.nih.gov/protein/688587046?report=genbank&log$=prottop&blast_rank=1&RID=73D8JR6X01R) | ENSP00000383303 |
| gabrb3 | 6 | 498 | 94 | 83 | 3 | 0 | [XP_005166137.1](http://www.ncbi.nlm.nih.gov/protein/528482985?report=genbank&log$=prottop&blast_rank=1&RID=73D8JR6X01R) | ENSP00000442408 |
| grin2ba | 3 | 1353 | 87 | 63 | 1 | 0 | [XP_009297288.1](http://www.ncbi.nlm.nih.gov/protein/688540243?report=genbank&log$=prottop&blast_rank=1&RID=75ZKZFYH01R) | ENSP00000477455 |
| grin2bb | 1 | 1770 | - | 41 | 1 | 0 | [ENSDART00000047094](http://useast.ensembl.org/Danio_rerio/Transcript/Sequence_Protein?db=core;g=ENSDARG00000079348;r=3:1538000-1547700;t=ENSDART00000047094) | ENSP00000477455 |
| katnal2 | 10 | 485 | 83 | 67 | 1 | 2.00E-177 | ENSDARP00000064900 | ENSP00000245121 |
| kdm5ba | 8 | 1383 | 98 | 61 | 2 | 0 | [XP_009302265.1](http://www.ncbi.nlm.nih.gov/protein/688567965?report=genbank&log$=prottop&blast_rank=3&RID=73D8JR6X01R) | ENSP00000356233 |
| kdm5bb | 11 | 1503 | 98 | 62 | 5 | 0 | [NP_001002166.1](http://www.ncbi.nlm.nih.gov/protein/50344988?report=genbank&log$=prottop&blast_rank=1&RID=73D8JR6X01R) | ENSP00000356233 |
| kmt2ca | 24 | 4942 | 99 | 49 | 4 | 0 | [XP_005162604.1](http://www.ncbi.nlm.nih.gov/protein/528519285?report=genbank&log$=prottop&blast_rank=1&RID=73D8JR6X01R) | ENSP00000262189 |
| kmt2cb | 2 | 4880 | 93 | 45 | 6 | 0 | [XP_005163354.1](http://www.ncbi.nlm.nih.gov/protein/528470515?report=genbank&log$=prottop&blast_rank=5&RID=73D8JR6X01R) | ENSP00000262189 |
| mecp2 | 8 | 523 | 83 | 48 | 3 | 2.00E-96 | [XP_005166744.1](http://www.ncbi.nlm.nih.gov/protein/528486824?report=genbank&log$=prottop&blast_rank=1&RID=FN9G6W2401R) | ENSP00000395535 |
| med13a | 10 | 2175 | - | 49 | 4 | 0 | [ENSDARP00000083285](http://useast.ensembl.org/Danio_rerio/Transcript/ProteinSummary?db=core;t=ENSDARP00000083285;tl=MRK4uhARfuCpAK0B-1341887-292187163) | ENSP00000380888 |
| med13b | 15 | 2102 | 99 | 52 | 2 | 0 | NP_001268397.1 | ENSP00000380888 |
| met | 25 | 1382 | 98 | 51 | 2 | 0 | [XP_005163051.1](http://www.ncbi.nlm.nih.gov/protein/528521414?report=genbank&log$=prottop&blast_rank=1&RID=73D8JR6X01R) | ENSP00000317272 |
| myt1la | 20 | 1236 | 83 | 69 | 3 | 0 | [XP_005160780.1](http://www.ncbi.nlm.nih.gov/protein/528511783?report=genbank&log$=prottop&blast_rank=2&RID=73D8JR6X01R) | ENSP00000382114 |
| ndnl2 (magel) | 23 | 260 | 16 | 48 | 2 |  | [NP_001269019.1](http://www.ncbi.nlm.nih.gov/protein/530719564?report=genbank&log$=prottop&blast_rank=1&RID=73D8JR6X01R) | [NM_138704.3](http://www.ncbi.nlm.nih.gov/nuccore/332078556) |
| nrxn1a | 12 | 1454 | 99 | 78 | 17 | 0 | [XP_009304929.1](http://www.ncbi.nlm.nih.gov/protein/688579865?report=genbank&log$=prottop&blast_rank=1&RID=73D8JR6X01R) | ENSP00000385017 |
| nrxn1b | 13 | 1484 | - | 65 | 5 | 0 | [ENSDARP00000123742](http://useast.ensembl.org/Danio_rerio/Transcript/ProteinSummary?db=core;t=ENSDARP00000123742;tl=Aw0LKHMVFbN7ruXM-1343956-292563132) | ENSP00000385017 |
| ptchd1 | 24 | 887 | 99 | 71 | 1 | 0 | XP_690754.1 | ENSP00000368666 |
| ptena | 17 | 431 | 99 | 83 | 4 | 0 | [XP_009291324.1](http://www.ncbi.nlm.nih.gov/protein/688594467?report=genbank&log$=prottop&blast_rank=1&RID=75ZKZFYH01R) | ENSP00000489359 |
| ptenb | 12 | 399 | 99 | 87 | 2 | 0 | [NP_001001822.1](http://www.ncbi.nlm.nih.gov/protein/49227345?report=genbank&log$=prottop&blast_rank=2&RID=75ZKZFYH01R) | ENSP00000489359 |
| reln | 18 | 3468 | 99 | 68 | 2 | 0 | [NP_001233205.1](http://www.ncbi.nlm.nih.gov/protein/350536581?report=genbank&log$=prottop&blast_rank=1&RID=73D8JR6X01R) | RELN\|ENSP00000345694 |
| setd5 | 6 | 1471 | 81 | 50 | 3 | 0 | [XP_698834.4](http://www.ncbi.nlm.nih.gov/protein/292614856?report=genbank&log$=prottop&blast_rank=1&RID=75ZKZFYH01R) | ENSP00000384114 |
| slc9a6a | 14 | 697 | 97 | 78 | 2 | 0 | [NP_001091726.2](http://www.ncbi.nlm.nih.gov/protein/218505672?report=genbank&log$=prottop&blast_rank=1&RID=FN9G6W2401R) | ENSP00000359732 |
| slc9a6b | 10 | 692 | 97 | 74 | 1 | 0 | [NP_001106947.1](http://www.ncbi.nlm.nih.gov/protein/164698430?report=genbank&log$=prottop&blast_rank=3&RID=FN9G6W2401R) | ENSP00000359732 |
| suv420h1 | 18 | 808 | 99 | 54 | 1 | 0 | NP_001007338.1 | ENSP00000305899 |
| znf292a | 17 | 2313 | 58 | 51 | 4 | 0 | [NP_001098611.2](http://www.ncbi.nlm.nih.gov/protein/939320298?report=genbank&log$=prottop&blast_rank=2&RID=FN9G6W2401R) | ENSP00000358590 |
| znf292b | 20 | 2619 | 65 | 57 | 2 | 0 | [NP_001025383.1](http://www.ncbi.nlm.nih.gov/protein/71834564?report=genbank&log$=prottop&blast_rank=1&RID=FN9G6W2401R) | ENSP00000358590 |
|  | Md | 1334.5 |  |  |  |  |  |  |
|  | SEM | 227 |  |  |  |  |  |  |

**Table 2. Autism Spectrum Disorder-Intellectual Disability Gene Orthologues Blast results. Ch., chromosome; aa, protein length in protein length in amino acids; Iso., isoforms (ENSMBL.org/NCBI.gov).**

| **Gene** | **Ch** | **aa** | **Query Cover %** | **% Protein ID** | **Iso.** | **E-Value** | **Zebrafish Protein NCBI/ENSEMBL ID** | **Human Protein NCBI/ENSEMBL ID** |
| --- | --- | --- | --- | --- | --- | --- | --- | --- |
| adnpa | 11 | 969 | 93 | 52 | 2 | 0 | [NP_001073484.1](http://www.ncbi.nlm.nih.gov/protein/121583645?report=genbank&log$=prottop&blast_rank=3&RID=75ZKZFYH01R) | ENSP00000342905\|ADNP |
| adnpb | 23 | 1016 | 77 | 61 | 2 |  | [XP_009295051.1](http://www.ncbi.nlm.nih.gov/protein/688611437?report=genbank&log$=prottop&blast_rank=1&RID=75ZKZFYH01R) | ENSP00000342905\|ADNP |
| arhgef6 | 14 | 766 | 98 | 64 | 2 | 0.00E+00 | [XP_686581.4](http://www.ncbi.nlm.nih.gov/protein/326673680?report=genbank&log$=prottop&blast_rank=1&RID=9DDADSUK013) | ENSP00000250617\|ARHGEF6 |
| arid1b | 20 | 2122 | 94 | 50 | 6 |  | [XP_698079.4](http://www.ncbi.nlm.nih.gov/protein/528510950?report=genbank&log$=prottop&blast_rank=1&RID=75ZKZFYH01R) | ENSP00000344546\|ARID1B |
| auts2 | 10 | 1259 |  |  |  |  | [F1QFN2](http://www.uniprot.org/uniprot/F1QFN2) | ENSP00000344087\|AUTS2 |
| cdh15 | 7 | 792 | 92 | 45 | 2 | 0 | [NP_997771.2](http://www.ncbi.nlm.nih.gov/protein/940373623?report=genbank&log$=prottop&blast_rank=1&RID=9DDADSUK013) | ENSP00000289746\|CDH15 |
| dyrk1aa | 10 | 719 | 96 | 78 | 5 | 0 | [XP_002664656.2](http://www.ncbi.nlm.nih.gov/protein/528501612?report=genbank&log$=prottop&blast_rank=1&RID=75ZKZFYH01R) | ENSP00000381932\|DYK1A |
| dyrk1ab | 15 | 737 | 99 | 82 | 3 | 0 | [NP_001074158.1](http://www.ncbi.nlm.nih.gov/protein/123701398?report=genbank&log$=prottop&blast_rank=2&RID=75ZKZFYH01R) | ENSP00000381932\|DYK1A |
| fmr1 | 14 | 569 | 90 | 80 | 9 | 0.00E+00 | [NP_694495.1](http://www.ncbi.nlm.nih.gov/protein/23308667?report=genbank&log$=prottop&blast_rank=1&RID=9UN35787015) | ENSP00000218200\|FMR1 |
| foxp1a | 23 | 663 |  | 68 | 3 |  | [ENSDARP00000107924](http://useast.ensembl.org/Danio_rerio/Transcript/ProteinSummary?db=core;t=ENSDARP00000107924;tl=MRK4uhARfuCpAK0B-1341893-292187087) | ENSP00000318902\|FOXP1 |
| foxp1b | 6 | 660 | 99 | 76 | 4 | 0 | [XP_005166114.1](http://www.ncbi.nlm.nih.gov/protein/528482936?report=genbank&log$=prottop&blast_rank=1&RID=73D8JR6X01R) | ENSP00000318902\|FOXP1 |
| huwe1 | 23 | 4556 | 99 | 77 | 6 | 0.00E+00 | [XP_001923900.2](http://www.ncbi.nlm.nih.gov/protein/528518229?report=genbank&log$=prottop&blast_rank=1&RID=9DDADSUK013) | ENSP00000262854\|HUWE1 |
| il1rapl1a | 9 | 701 | 99 | 68 | 2 | 0 | [B6ZK76.1](http://www.ncbi.nlm.nih.gov/protein/284433480?report=genbank&log$=prottop&blast_rank=2&RID=9DDADSUK013) | ENSP00000368278\|IL1RAPL1 |
| il1rapl1b | 11 | 700 | 99 | 75 | 1 | 0 | [NP_001136054.1](http://www.ncbi.nlm.nih.gov/protein/217272826?report=genbank&log$=prottop&blast_rank=1&RID=9DDADSUK013) | ENSP00000368278\|IL1RAPL1 |
| kirrel3a | 10 | 753 | 85 | 75 | 2 | 0 | [XP_009303878.1](http://www.ncbi.nlm.nih.gov/protein/688575348?report=genbank&log$=prottop&blast_rank=1&RID=9DDADSUK013) | ENSP00000435094\|KIRREL3 |
| kirrel3b | 15 | 769 | 87 | 53 | 1 | 0 | [CAP71967.1](http://www.ncbi.nlm.nih.gov/protein/165993305?report=genbank&log$=prottop&blast_rank=4&RID=9DDADSUK013) | ENSP00000435094\|KIRREL3 |
| nlgn4a | 1 | 826 | 99 | 82 | 3 | 0 | [XP_009290449.1](http://www.ncbi.nlm.nih.gov/protein/688533043?report=genbank&log$=prottop&blast_rank=1&RID=9DDADSUK013) | ENSP00000275857\|NLGN4X |
| nlgn4b | 9 | 795 | 95 | 79 | 1 | 0 | [XP_009298767.1](http://www.ncbi.nlm.nih.gov/protein/688572755?report=genbank&log$=prottop&blast_rank=6&RID=9DDADSUK013) | ENSP00000275857\|NLGN4X |
| ophn1 | 5 | 805 | 74 | 61 | 4 | 0 | [Q6ZM89.3](http://www.ncbi.nlm.nih.gov/protein/727863585?report=genbank&log$=prottop&blast_rank=1&RID=9DDADSUK013) | ENSP00000347710\|OPHN1 |
| pak3 | 19 | 492 | 50 | 43 | 1 | 1.00E-74 | [NP_955966.1](http://www.ncbi.nlm.nih.gov/protein/41054445?report=genbank&log$=prottop&blast_rank=15&RID=9DDADSUK013) | ENSP00000262836\|PAK3 |
| pogza | 19 | 1277 | 99 | 38 | 1 | 0 | [NP_001201839.1](http://www.ncbi.nlm.nih.gov/protein/333805607?report=genbank&log$=prottop&blast_rank=2&RID=75ZKZFYH01R) | ENSP00000271715\|POGZ |
| pogzb | 16 | 1373 | 95 | 39 | 4 | 0 | [XP_003200290.1](http://www.ncbi.nlm.nih.gov/protein/326675150?report=genbank&log$=prottop&blast_rank=1&RID=75ZKZFYH01R) | ENSP00000271715\|POGZ |
| scn1lab | 6 | 1996 | 99 | 79 | 3 | 0 | [XP_009300462.1](http://www.ncbi.nlm.nih.gov/protein/688558946?report=genbank&log$=prottop&blast_rank=1&RID=75ZKZFYH01R) | ENSP00000364586\|SCN2A |
| scn1a | 9 | 1956 | 99 | 67 | 11 | 0 | [NP_956426.2](http://www.ncbi.nlm.nih.gov/protein/103471981?report=genbank&log$=prottop&blast_rank=14&RID=NSY2NMR6014) | ENSP00000364586\|SCN2A |
| shank2 | 25 | 1235 | 91 | 71 | 5 | 0 | [XP_009296195.1](http://www.ncbi.nlm.nih.gov/protein/688616549?report=genbank&log$=prottop&blast_rank=1&RID=73D8JR6X01R) | ENSP00000469689\|SHANK2 |
| shank3a | 18 | 1552 | 82 | 58 | 6 | 0 | [XP_009291714.1](http://www.ncbi.nlm.nih.gov/protein/688596229?report=genbank&log$=prottop&blast_rank=1&RID=765RZBGN01R) | ENSP00000489147\|SHANK3 |
| shank3b | 4 | 1605 | 63 | 63 | 6 |  | [XP_009298462.1](http://www.ncbi.nlm.nih.gov/protein/688547143?report=genbank&log$=prottop&blast_rank=2&RID=765RZBGN01R) | ENSP00000489147\|SHANK3 |
| stxbp1a | 21 | 603 | 99 | 88 | 3 | 0 | [XP_005161170.1](http://www.ncbi.nlm.nih.gov/protein/528513367?report=genbank&log$=prottop&blast_rank=1&RID=9DDADSUK013) | ENSP00000362399\|STXBP1 |
| stxbp1b | 5 | 605 | 99 | 78 | 1 | 0 | [NP_001082845.1](http://www.ncbi.nlm.nih.gov/protein/148232800?report=genbank&log$=prottop&blast_rank=3&RID=9DDADSUK013) | ENSP00000362399\|STXBP1 |
| syngap1a | 19 | 1311 | 97 | 61 | 3 | 0 | [XP_003200549.2](http://www.ncbi.nlm.nih.gov/protein/528508795?report=genbank&log$=prottop&blast_rank=2&RID=75ZKZFYH01R) | ENSP00000378509\|SYNGAP1 |
| syngap1b | 16 | 1413 | 97 | 61 | 9 | 0 | [XP_009290924.1](http://www.ncbi.nlm.nih.gov/protein/688592685?report=genbank&log$=prottop&blast_rank=1&RID=75ZKZFYH01R) | ENSP00000378509\|SYNGAP1 |
| sypa | 8 | 297 | 97 | 69 | 2 | 9.00E-144 | [NP_001137449.1](http://www.ncbi.nlm.nih.gov/protein/221136800?report=genbank&log$=prottop&blast_rank=1&RID=9DDADSUK013) | ENSP00000263233\|SYP |
| sypb | 8 | 297 | 97 | 63 | 4 | 1.00E-123 | [NP_001025413.2](http://www.ncbi.nlm.nih.gov/protein/221139918?report=genbank&log$=prottop&blast_rank=4&RID=9DDADSUK013) | ENSP00000263233\|SYP |
| tbr1a | 11 | 676 | 99 | 77 | 1 | 0 | [AAG48249.1](http://www.ncbi.nlm.nih.gov/protein/12060814?report=genbank&log$=prottop&blast_rank=2&RID=75ZKZFYH01R) | ENSP00000374205\|TBR1 |
| tbr1b | 9 | 676 | 99 | 77 | 1 | 0 | [NP_001108562.1](http://www.ncbi.nlm.nih.gov/protein/169259786?report=genbank&log$=prottop&blast_rank=1&RID=75ZKZFYH01R) | ENSP00000374205\|TBR1 |
|  | Md | 781 |  |  |  |  |  |  |
|  | SEM | 210 |  |  |  |  |  |  |

**Table 3. Charcot-Marie-Tooth Gene Orthologues BLAST results. Ch., chromosome; aa, protein length in protein length in amino acids; Iso., isoforms (ENSMBL.org/NCBI.gov).**

| **Gene** | **Ch.** | **aa** | **Query Cover %** | **% Protein ID** | **Iso.** | **E-Value** | **Zebrafish Protein NCBI/ENSEMBL ID** | **Human Protein NCBI/ENSEMBL ID** |
| --- | --- | --- | --- | --- | --- | --- | --- | --- |
| dnm2a | 3 | 860 | 99 | 86 | 6 | 0 | [XP_005164298.1](http://www.ncbi.nlm.nih.gov/protein/528475019?report=genbank&log$=prottop&blast_rank=1&RID=F7XZVNZ4014) | ENSP00000347890\|DNM2 |
| dnm2l | 25 | 856 | 99 | 82 | 2 | 0 | [NP_998407.1](http://www.ncbi.nlm.nih.gov/protein/47086061?report=genbank&log$=prottop&blast_rank=7&RID=F7XZVNZ4014) | ENSP00000347890\|DNM2 |
| egr2a | 17 | 393 | 89 | 52 | 4 | 2.00E-129 | [XP_005158926.1](http://www.ncbi.nlm.nih.gov/protein/528506798?report=genbank&log$=prottop&blast_rank=3&RID=F7XZVNZ4014) | ENSP00000242480\|EGR2 |
| egr2b | 12 | 412 | 89 | 66 | 2 | 1.00E-178 | [NP_571072.2](http://www.ncbi.nlm.nih.gov/protein/52138558?report=genbank&log$=prottop&blast_rank=1&RID=F7XZVNZ4014) | ENSP00000242480\|EGR2 |
| fgd4a | 18 | 728 | 72 | 73 | 1 | 0 | [NP_001171404.1](http://www.ncbi.nlm.nih.gov/protein/295789009?report=genbank&log$=prottop&blast_rank=1&RID=F7XZVNZ4014) | ENSP00000394487\|FGD4 |
| fgd4l | 4 | 653 | 73 | 68 | 1 | 0 | [XP_001332264.5](http://www.ncbi.nlm.nih.gov/protein/528475750?report=genbank&log$=prottop&blast_rank=2&RID=F7XZVNZ4014) | ENSP00000394487\|FGD4 |
| gars | 6 | 779 | 95 | 85 | 1 | 0 | [XP_009295729.1](http://www.ncbi.nlm.nih.gov/protein/688614330?report=genbank&log$=prottop&blast_rank=1&RID=F7XZVNZ4014) | ENSP00000373918\|GARS |
| gdap1 | 24 | 362 | 89 | 79 | 2 | 0 | [NP_001018511.1](http://www.ncbi.nlm.nih.gov/protein/66472398?report=genbank&log$=prottop&blast_rank=1&RID=F7XZVNZ4014) | ENSP00000220822\|GDAP1 |
| gdapl1 | 23 | 280 | 76 | 78 | 2 | 9.00E-159 | [XP_005162700.1](http://www.ncbi.nlm.nih.gov/protein/528519499?report=genbank&log$=prottop&blast_rank=2&RID=F7XZVNZ4014) | ENSP00000220822\|GDAP1 |
| gjb1 | 5 | 240 | 77 | 72 | 1 | 3.00E-123 | [NP_571886.1](http://www.ncbi.nlm.nih.gov/protein/18858493?report=genbank&log$=prottop&blast_rank=2&RID=F7XZVNZ4014) | ENSP00000363141\|GJB1 |
| gjb1l | 14 | 275 | 99 | 64 | 1 | 2.00E-131 | [XP_001921623.4](http://www.ncbi.nlm.nih.gov/protein/528499486?report=genbank&log$=prottop&blast_rank=1&RID=F7XZVNZ4014) | ENSP00000363141\|GJB1 |
| hint1 | 10 | 126 | 99 | 73 | 1 | 3.00E-68 | [NP_001005593.1](http://www.ncbi.nlm.nih.gov/protein/53933250?report=genbank&log$=prottop&blast_rank=1&RID=F7XZVNZ4014) | ENSP00000304229\|HINT1 |
| hsbp1 | 5 | 199 | 89 | 64 | 1 | 6.00E-83 | [NP_001008615.2](http://www.ncbi.nlm.nih.gov/protein/339717158?report=genbank&log$=prottop&blast_rank=1&RID=F7XZVNZ4014) | ENSP00000248553\|HSPB1 |
| hsbpl1 | 5 | 365 | 85 | 47 | 1 | 1.00E-43 | [XP_002662020.1](http://www.ncbi.nlm.nih.gov/protein/292613658?report=genbank&log$=prottop&blast_rank=2&RID=F7XZVNZ4014) | ENSP00000248553\|HSPB1 |
| inf2 | 17 | 1003 | 34 | 60 | 2 | 6.00E-151 | [XP_009291036.1](http://www.ncbi.nlm.nih.gov/protein/688593273?report=genbank&log$=prottop&blast_rank=1&RID=F7XZVNZ4014) | ENSP00000376406\|INF2 |
| litaf | 3 | 162 | 35 | 40 | 6 | 1.00E-11 | [XP_009297979.1](http://www.ncbi.nlm.nih.gov/protein/688544360?report=genbank&log$=prottop&blast_rank=1&RID=F7XZVNZ4014) | ENSP00000371231\|LITAF |
| mfn2 | 8 | 757 | 99 | 83 | 1 | 0 | [NP_001121726.1](http://www.ncbi.nlm.nih.gov/protein/190194317?report=genbank&log$=prottop&blast_rank=1&RID=F7XZVNZ4014) | ENSP00000235329\|MFN2 |
| mpz | 2 | 203 | 71 | 51 | 1 | 4.00E-54 | [NP_919342.1](http://www.ncbi.nlm.nih.gov/protein/35902687?report=genbank&log$=prottop&blast_rank=1&RID=F7XZVNZ4014) | ENSP00000431538\|MPZ |
| mtmr2 | 5 | 620 | 97 | 73 | 1 | 0 | [NP_571446.1](http://www.ncbi.nlm.nih.gov/protein/117606281?report=genbank&log$=prottop&blast_rank=1&RID=F7XZVNZ4014) | ENSP00000345752\|MTMR2 |
| nefla | 21 | 712 | 77 | 61 | 1 | 1.00E-174 | [XP_009293645.1](http://www.ncbi.nlm.nih.gov/protein/688605386?report=genbank&log$=prottop&blast_rank=2&RID=F7XZVNZ4014) | ENSP00000482169\|NEFL |
| neflb | 8 | 579 | 86 | 58 | 1 | 5.40E-177 | [NP_001034927.1](http://www.ncbi.nlm.nih.gov/protein/89886345?report=genbank&log$=prottop&blast_rank=1&RID=F7XZVNZ4014) | ENSP00000482169\|NEFL |
| ntrk1 | 16 | 797 | 94 | 55 | 2 | 0 | [NP_001288285.1](http://www.ncbi.nlm.nih.gov/protein/672349270?report=genbank&log$=prottop&blast_rank=1&RID=F7XZVNZ4014) | ENSP00000376120\|NTRK1 |
| pmp22a | 3 | 157 | 99 | 51 | 3 | 5.00E-32 | [NP_958468.1](http://www.ncbi.nlm.nih.gov/protein/41152006?report=genbank&log$=prottop&blast_rank=1&RID=F7XZVNZ4014) | ENSP00000462782\|PMP22 |
| pmp22b | 12 | 155 | 86 | 63 | 1 | 7.00E-30 | [NP_001035445.1](http://www.ncbi.nlm.nih.gov/protein/94536653?report=genbank&log$=prottop&blast_rank=3&RID=F7XZVNZ4014) | ENSP00000462782\|PMP22 |
| sptlc1 | 10 | 472 | 98 | 85 | 3 | 0 | [NP_001018307.1](http://www.ncbi.nlm.nih.gov/protein/66472918?report=genbank&log$=prottop&blast_rank=1&RID=F7XZVNZ4014) | ENSP00000262554\|SPTLC1 |
| trpv4 | 5 | 859 | 94 | 73 | 4 | 0 | [XP_005165208.1](http://www.ncbi.nlm.nih.gov/protein/528478882?report=genbank&log$=prottop&blast_rank=1&RID=F7XZVNZ4014) | ENSP00000261740\|TRPV4 |
| wink1a | 25 | 1606 | 41 | 67 | 9 | 0 | [XP_002666892.3](http://www.ncbi.nlm.nih.gov/protein/528521633?report=genbank&log$=prottop&blast_rank=13&RID=F7XZVNZ4014) | ENSP00000433548\|WNK1 |
| wink1b | 4 | 2418 | 81 | 56 | 11 | 0 | [XP_009298366.1](http://www.ncbi.nlm.nih.gov/protein/688546603?report=genbank&log$=prottop&blast_rank=1&RID=F7XZVNZ4014) | ENSP00000433548\|WNK1 |
|  | Md | 525.5 |  |  |  |  |  |  |
|  | SEM | 112 |  |  |  |  |  |  |

**Table 4. Hereditary Cerebellar Ataxia Gene Orthologues Blast results. Ch., chromosome; aa, protein length in amino acids; Iso., isoforms (ENSMBL.org/NCBI.gov).**

| **Gene** | **Ch.** | **aa** | **Query Cover %** | **% Protein ID** | **Iso.** | **E-Value** | **Zebrafish Protein NCBI/ENSEMBL ID** | **Human Protein NCBI/ENSEMBL ID** |
| --- | --- | --- | --- | --- | --- | --- | --- | --- |
| abcb7 | 14 | 743 | 91 | 75 | 1 | 0.00E+00 | [XP_694879.2](http://www.ncbi.nlm.nih.gov/protein/125834513?report=genbank&log$=prottop&blast_rank=1&RID=9UN35787015) | ENSP00000343849\|ABCB7 |
| afg3l2 | 2 | 800 | 99 | 81 | 1 | 0.00E+00 | [NP_001104667.1](http://www.ncbi.nlm.nih.gov/protein/162287285?report=genbank&log$=prottop&blast_rank=1&RID=9RAY0X4Y015) | ENSP00000269143\|AFG3L2 |
| ano10a | 16 | 646 | 97 | 66 | 2 | 0.00E+00 | [NP_001025377.1](http://www.ncbi.nlm.nih.gov/protein/71834552?report=genbank&log$=prottop&blast_rank=1&RID=9UAWYBH1014) | ENSP00000292246\|ANO10 |
| ano10b | 18 | 691 | 95 | 41 | 3 | 8.00E-163 | [XP_005174389.1](http://www.ncbi.nlm.nih.gov/protein/528521306?report=genbank&log$=prottop&blast_rank=2&RID=9UAWYBH1014) | ENSP00000292246\|ANO10 |
| aptx | 1 | 324 | 94 | 58 | 3 | 5.00E-135 | [NP_999894.1](http://www.ncbi.nlm.nih.gov/protein/47550739?report=genbank&log$=prottop&blast_rank=1&RID=9UAWYBH1014) | ENSP00000369147\|APTX |
| atm | 15 | 2773 | 89 | 53 | 2 | 0.00E+00 | [BAD91491.1](http://www.ncbi.nlm.nih.gov/protein/62084152?report=genbank&log$=prottop&blast_rank=1&RID=9UAWYBH1014) | ENSP00000278616\|ATM |
| atn1 | 16 | 1666 | 24 | 64 | 3 | 1.00E-31 | [XP_005158246.1](http://www.ncbi.nlm.nih.gov/protein/528504307?report=genbank&log$=prottop&blast_rank=7&RID=9RAY0X4Y015) | ENSP00000379915\|ATN1 |
| atxn1 | 19 | 781 | 99 | 43 | 3 | 2.00E-171 | [NP_001038291.1](http://www.ncbi.nlm.nih.gov/protein/113678036?report=genbank&log$=prottop&blast_rank=1&RID=9RAY0X4Y015) | ENSP00000244769\|ATXN1 |
| atxn10 | 4 | 484 | 94 | 39 | 1 | 3.00E-107 | [NP_001124084.1](http://www.ncbi.nlm.nih.gov/protein/194578853?report=genbank&log$=prottop&blast_rank=1&RID=9RAY0X4Y015) | ENSP00000252934\|ATXN10 |
| atxn2 | 5 | 1112 | 99 | 62 | 1 | 0.00E+00 | [NP_001121821.1](http://www.ncbi.nlm.nih.gov/protein/190358425?report=genbank&log$=prottop&blast_rank=2&RID=9RAY0X4Y015) | ENSP00000373805\|ATXN2 |
| atxn3 | 17 | 306 | 79 | 72 | 2 | 5.00E-150 | [AAY28605.1](http://www.ncbi.nlm.nih.gov/protein/63053592?report=genbank&log$=prottop&blast_rank=1&RID=9RAY0X4Y015) | ENSP00000478320\|ATXN3 |
| atxn7 | 11 | 866 | 99 | 46 | 3 | 0.00E+00 | [XP_009304240.1](http://www.ncbi.nlm.nih.gov/protein/688576997?report=genbank&log$=prottop&blast_rank=1&RID=9RAY0X4Y015) | ENSP00000295900\|ATXN7 |
| bean1 | 7 | 282 | 84 | 40 | 2 | 8.00E-33 | [XP_009301679.1](http://www.ncbi.nlm.nih.gov/protein/688565158?report=genbank&log$=prottop&blast_rank=1&RID=9RAY0X4Y015) | ENSP00000442793\|BEAN1 |
| c12h10orf2 | 12 | 728 | 88 | 65 | 1 | 0.00E+00 | [NP_001264527.1](http://www.ncbi.nlm.nih.gov/protein/478431081?report=genbank&log$=prottop&blast_rank=1&RID=9UAWYBH1014) | ENSP00000309595\|C10orf2 |
| cacna1aa | 3 | 2343 | 96 | 71 | 12 | 0.00E+00 | [XP_009297869.1](http://www.ncbi.nlm.nih.gov/protein/688543825?report=genbank&log$=prottop&blast_rank=1&RID=9RAY0X4Y015) | ENSP00000353362\|CACNA1A |
| cacna1ab | 11 | 2458 | 99 | 67 | 9 | 0.00E+00 | [XP_009304410.1](http://www.ncbi.nlm.nih.gov/protein/688577654?report=genbank&log$=prottop&blast_rank=11&RID=9RAY0X4Y015) | ENSP00000353362\|CACNA1A |
| ccdc88c | 17 | 1997 | 99 | 53 | 4 | 0.00E+00 | [XP_001921927.1](http://www.ncbi.nlm.nih.gov/protein/189531040?report=genbank&log$=prottop&blast_rank=1&RID=9RAY0X4Y015) | ENSP00000374507\|CCDC88C |
| cyp27a | 9 | 522 | 90 | 48 | 4.00E+00 | 8.00E-165 | [XP_001334004.3](http://www.ncbi.nlm.nih.gov/protein/292617767?report=genbank&log$=prottop&blast_rank=1&RID=9UAWYBH1014) | ENSP00000258415\|CYP27A1 |
| dnmt1 | 3 | 1500 | 94 | 75 | 1 | 0.00E+00 | [AAI63894.1](http://www.ncbi.nlm.nih.gov/protein/190338613?report=genbank&log$=prottop&blast_rank=1&RID=9RAY0X4Y015) | ENSP00000345739\|DNMT1 |
| eef2b | 2 | 858 | 99 | 93 | 1 | 0.00E+00 | [NP_956752.2](http://www.ncbi.nlm.nih.gov/protein/41386743?report=genbank&log$=prottop&blast_rank=2&RID=9RAY0X4Y015) | ENSP00000307940\|EEF2 |
| eefl2 | 5 | 861 | 99 | 92 | 1 | 0.00E+00 | [XP_697966.5](http://www.ncbi.nlm.nih.gov/protein/528480064?report=genbank&log$=prottop&blast_rank=1&RID=9RAY0X4Y015) | ENSP00000307940\|EEF2 |
| elovl4a | 16 | 309 | 90 | 68 | 2 | 7.00E-150 | [NP_957090.1](http://www.ncbi.nlm.nih.gov/protein/41387170?report=genbank&log$=prottop&blast_rank=1&RID=9RAY0X4Y015) | ENSP00000358831\|ELOVL4 |
| elovl4b | 23 | 287 | 92 | 67 | 2 | 6.00E-149 | [AAI52204.1](http://www.ncbi.nlm.nih.gov/protein/156230054?report=genbank&log$=prottop&blast_rank=2&RID=9RAY0X4Y015) | ENSP00000358831\|ELOVL4 |
| elovl5 | 13 | 291 | 99 | 81 |  | 9.00E-157 | [NP_956747.1](http://www.ncbi.nlm.nih.gov/protein/41055213?report=genbank&log$=prottop&blast_rank=1&RID=9RAY0X4Y015) | ENSP00000306640\|ELOVL5 |
| fgf14 | 9 | 242 | 82 | 92 | 2 | 0.00E+00 | [XP_009303010.1](http://www.ncbi.nlm.nih.gov/protein/688571376?report=genbank&log$=prottop&blast_rank=1&RID=9RAY0X4Y015) | ENSP00000365301\|FGF14 |
| fmr1 | 14 | 569 | 90 | 80 | 9 | 0.00E+00 | [NP_694495.1](http://www.ncbi.nlm.nih.gov/protein/23308667?report=genbank&log$=prottop&blast_rank=1&RID=9UN35787015) | ENSP00000218200\|FMR1 |
| fxn | 8 | 169 | 61 | 63 | 1 | 7.00E-54 | [NP_001076485.1](http://www.ncbi.nlm.nih.gov/protein/131889783?report=genbank&log$=prottop&blast_rank=1&RID=9UAWYBH1014) | ENSP00000366482\|FXN |
| grid2 | 8 | 1009 | 99 | 82 | 5 | 0 | [AAI62459.1](http://www.ncbi.nlm.nih.gov/protein/190339228?report=genbank&log$=prottop&blast_rank=1&RID=9RAY0X4Y015) | ENSP00000282020\|GRID2 |
| itpr1a | 6 | 2710 | 99 | 81 | 5 | 0.00E+00 | [XP_009300860.1](http://www.ncbi.nlm.nih.gov/protein/688561322?report=genbank&log$=prottop&blast_rank=17&RID=9RAY0X4Y015) | ENSP00000346595\|ITPR1 |
| itpr1b | 11 | 2762 | 99 | 85 | 16 | 0.00E+00 | [XP_009304486.1](http://www.ncbi.nlm.nih.gov/protein/688577880?report=genbank&log$=prottop&blast_rank=1&RID=9RAY0X4Y015) | ENSP00000346595\|ITPR1 |
| kcnc3a | 3 | 609 | 99 | 61 | 5 | 0.00E+00 | [XP_009297824.1](http://www.ncbi.nlm.nih.gov/protein/688543551?report=genbank&log$=prottop&blast_rank=2&RID=9RAY0X4Y015) | ENSP00000366158\|KCNC3 |
| kcnc3b | 24 | 647 | 87 | 67 | 2 | 0.00E+00 | [NP_001182170.1](http://www.ncbi.nlm.nih.gov/protein/305410803?report=genbank&log$=prottop&blast_rank=1&RID=9RAY0X4Y015) | ENSP00000366158\|KCNC3 |
| kcnd3 | 8 | 638 | 99 | 77 | 3 | 0.00E+00 | [NP_956096.1](http://www.ncbi.nlm.nih.gov/protein/41054215?report=genbank&log$=prottop&blast_rank=1&RID=9RAY0X4Y015) | ENSP00000306923\|KCND3 |
| nop56 | 21 | 366 | 79 | 89 | 1 | 2.00E-110 | [AAH90915.1](http://www.ncbi.nlm.nih.gov/protein/60551194?report=genbank&log$=prottop&blast_rank=1&RID=9RAY0X4Y015) | ENSP00000403199\|NOP56 |
| ophn1 | 5 | 701 | 62 | 58 | 3 | 0.00E+00 | [CAE47767.1](http://www.ncbi.nlm.nih.gov/protein/37359686?report=genbank&log$=prottop&blast_rank=4&RID=9UN35787015) | ENSP00000347710\|OPHN1 |
| pdyn | 23 | 261 | 98 | 41 | 4 | 7.00E-45 | [XP_009295380.1](http://www.ncbi.nlm.nih.gov/protein/688612697?report=genbank&log$=prottop&blast_rank=3&RID=9JTAS7XM01R) | ENSP00000217305\|PDYN |
|  | Md | 673 |  |  |  |  |  |  |
|  | SEM | 162 |  |  |  |  |  |  |

**Table 5. Hereditary Spastic Paraplegia Blast results. Ch., chromosome; aa, protein length in amino acids; Iso., isoforms (ENSMBL.org/NCBI.gov).**

| **Gene** | **Ch** | **aa** | **Query Cover %** | **% Protein ID** | **Iso.** | **E-Value** | **Zebrafish Protein NCBI/ENSEMBL ID** | **Human Protein NCBI/ENSEMBL ID** |
| --- | --- | --- | --- | --- | --- | --- | --- | --- |
| ap4b1 | 23 | 721 | 99 | 58 | 2 | 0.00E+00 | [NP_956632.1](http://www.ncbi.nlm.nih.gov/protein/41056081?report=genbank&log$=prottop&blast_rank=1&RID=9WWKB7NX014) | ENSP00000256658\|AP4B1 |
| ap4e1 | 25 | 1121 | 99 | 54 | 1 | 0.00E+00 | [XP_699042.2](http://www.ncbi.nlm.nih.gov/protein/125854498?report=genbank&log$=prottop&blast_rank=1&RID=9DDADSUK013) | ENSP00000261842\|AP4E1 |
| ap4m1 | 10 | 442 | 99 | 60 | 1 | 0 | [NP_001002672.1](http://www.ncbi.nlm.nih.gov/protein/50540412?report=genbank&log$=prottop&blast_rank=1&RID=9DDADSUK013) | ENSP00000352603\|AP4M1 |
| ap4s1 | 17 | 141 | 65 | 73 | 1 | 1.00E-54 | [XP_005158818.1](http://www.ncbi.nlm.nih.gov/protein/528506366?report=genbank&log$=prottop&blast_rank=1&RID=9DDADSUK013) | ENSP00000216366\|AP4S1 |
| atl1 | 13 | 559 | 99 | 80 | 2 | 0.00E+00 | [XP_005157066.1](http://www.ncbi.nlm.nih.gov/protein/528498512?report=genbank&log$=prottop&blast_rank=1&RID=9V8WZT5K015) | ENSP00000351155\|ATL1 |
| bscl2 | 21 | 350 | 74 | 51 | 3 | 9.00E-110 | [NP_001032473.1](http://www.ncbi.nlm.nih.gov/protein/82658214?report=genbank&log$=prottop&blast_rank=1&RID=9V8WZT5K015) | ENSP00000385332\|BSCL2 |
| bscl2l | 14 | 391 | 65 | 49 | 3 | 3.00E-97 | [XP_005173142.1](http://www.ncbi.nlm.nih.gov/protein/528499267?report=genbank&log$=prottop&blast_rank=3&RID=9V8WZT5K015) | ENSP00000385332\|BSCL2 |
| c18h19orf12 | 18 | 143 | 91 | 57 | 2 | 3.00E-34 | [XP_003199042.1](http://www.ncbi.nlm.nih.gov/protein/326669552?report=genbank&log$=prottop&blast_rank=1&RID=9WWKB7NX014) | ENSP00000376103\|C19orf12 |
| c5h12orf65 | 5 | 137 | 70 | 62 | 2 | 1.00E-43 | [XP_009300308.1](http://www.ncbi.nlm.nih.gov/protein/688558107?report=genbank&log$=prottop&blast_rank=1&RID=9WWKB7NX014) | ENSP00000253233\|C12orf65 |
| cx47.1 | 2 | 409 | 99 | 49 | 1 | 4.00E-137 | [NP_001004574.1](http://www.ncbi.nlm.nih.gov/protein/52219002?report=genbank&log$=prottop&blast_rank=1&RID=9WWKB7NX014) | ENSP00000355675\|GJC2 |
| ddhd1a | 17 | 793 | 81 | 65 | 2 | 0 | [XP_691327.4](http://www.ncbi.nlm.nih.gov/protein/326675812?report=genbank&log$=prottop&blast_rank=2&RID=9WWKB7NX014) | ENSP00000327104\|DDHD1 |
| ddhd1b | 20 | 861 | 84 | 62 | 2.00E+00 | 0.00E+00 | [CAK10968.1](http://www.ncbi.nlm.nih.gov/protein/94734055?report=genbank&log$=prottop&blast_rank=1&RID=9WWKB7NX014) | ENSP00000327104\|DDHD1 |
| erlin2 | 5 | 342 | 88 | 84 | 2 | 0.00E+00 | [NP_001121887.1](http://www.ncbi.nlm.nih.gov/protein/190358429?report=genbank&log$=prottop&blast_rank=1&RID=9WWKB7NX014) | ENSP00000276461\|ERLIN2 |
| fa2h | 18 | 377 | 22 | 56 |  | 1.00E-119 | [ENSDARP00000107067](http://useast.ensembl.org/Danio_rerio/Transcript/ProteinSummary?db=core;t=ENSDARP00000107067;tl=9H1VvsNfv5S7MLdX-1416901-319031689) | ENSP00000219368\|FA2H |
| gad1a | 9 | 521 | 96 | 81 | 2 | 0.00E+00 | [XP_002663350.1](http://www.ncbi.nlm.nih.gov/protein/292617433?report=genbank&log$=prottop&blast_rank=2&RID=9WWKB7NX014) | ENSP00000350928\|GAD1 |
| gad1b | 6 | 587 | 96 | 84 | 3 | 0.00E+00 | [NP_919400.1](http://www.ncbi.nlm.nih.gov/protein/35903113?report=genbank&log$=prottop&blast_rank=1&RID=9WWKB7NX014) | ENSP00000350928\|GAD1 |
| gba2 | 7 | 851 | 90 | 68 | 3 | 0 | [XP_687652.2](http://www.ncbi.nlm.nih.gov/protein/125821356?report=genbank&log$=prottop&blast_rank=1&RID=9WWKB7NX014) | ENSP00000367343\|GBA2 |
| hspd1 | 9 | 575 | 96 | 81 | 2 | 0.00E+00 | [NP_851847.1](http://www.ncbi.nlm.nih.gov/protein/31044489?report=genbank&log$=prottop&blast_rank=1&RID=9V8WZT5K015) | ENSP00000340019\|HSPD1 |
| kiaa0196 | 16 | 1159 | 99 | 88 | 1 | 0.00E+00 | [NP_956477.1](http://www.ncbi.nlm.nih.gov/protein/41055722?report=genbank&log$=prottop&blast_rank=1&RID=9V8WZT5K015) | ENSP00000318016\|KIAA0196 |
| kif1aa | 6 | 1809 | 99 | 80 | 21 | 0 | [XP_005166007.1](http://www.ncbi.nlm.nih.gov/protein/528482501?report=genbank&log$=prottop&blast_rank=1&RID=9WWKB7NX014) | ENSP00000438388\|KIF1A |
| kif1ab | 2 | 1790 | 99 | 77 | 20 | 0.00E+00 | [XP_009296796.1](http://www.ncbi.nlm.nih.gov/protein/688536921?report=genbank&log$=prottop&blast_rank=11&RID=9WWKB7NX014) | ENSP00000438388\|KIF1A |
| kif5aa | 9 | 1033 | 99 | 70 | 3 | 0.00E+00 | [NP_001186705.1](http://www.ncbi.nlm.nih.gov/protein/315138992?report=genbank&log$=prottop&blast_rank=1&RID=9V8WZT5K015) | ENSP00000286452\|KIF5A |
| kif5ab | 6 | 1023 | 97 | 65 | 3 | 0.00E+00 | [XP_001339650.5](http://www.ncbi.nlm.nih.gov/protein/528483603?report=genbank&log$=prottop&blast_rank=2&RID=9V8WZT5K015) | ENSP00000286452\|KIF5A |
| nipa1 | 6 | 306 | 87 | 62 | 1 | 2.00E-116 | [NP_001006062.1](http://www.ncbi.nlm.nih.gov/protein/54400626?report=genbank&log$=prottop&blast_rank=1&RID=9WPS1FJ8014) | NP_653200.2 |
| pnpla6 | 1 | 1343 | 99 | 73 | 4 | 0 | XP_005160109.1 | ENSP00000221249\|PNPLA6 |
| reep1 | 17 | 199 | 90 | 71 | 1 | 7.00E-88 | [XP_001340207.3](http://www.ncbi.nlm.nih.gov/protein/528506825?report=genbank&log$=prottop&blast_rank=1&RID=9V8WZT5K015) | ENSP00000438346\|REEP1 |
| rtn2a | 15 | 203 | 32 | 45 | 4 | 3.00E-46 | [NP_001025136.1](http://www.ncbi.nlm.nih.gov/protein/71480119?report=genbank&log$=prottop&blast_rank=24&RID=9V8WZT5K015) | ENSP00000245923\|RTN2 |
| rtn2b | 21 | 208 | 30 | 42 | 2 | 2.00E-38 | [AAI55733.1](http://www.ncbi.nlm.nih.gov/protein/161611687?report=genbank&log$=prottop&blast_rank=28&RID=9V8WZT5K015) | ENSP00000245923\|RTN2 |
| slc33a1 | 3 | 543 | 99 | 69 | 2 | 0.00E+00 | [NP_957402.1](http://www.ncbi.nlm.nih.gov/protein/41055797?report=genbank&log$=prottop&blast_rank=1&RID=9V8WZT5K015) | ENSP00000352456\|SLC33A1 |
| spast | 1 | 570 | 80 | 73 | 3 | [0](http://www.ncbi.nlm.nih.gov/protein/528469136?report=genbank&log$=prottop&blast_rank=1&RID=9V8WZT5K015) | [XP_005155454.1](http://www.ncbi.nlm.nih.gov/protein/528469136?report=genbank&log$=prottop&blast_rank=1&RID=9V8WZT5K015) | ENSP00000340817\|SPAST |
| tecpr2 | 17 | 1358 | 72 | 68 | 4 | 0.00E+00 | [XP_005158848.1](http://www.ncbi.nlm.nih.gov/protein/528506426?report=genbank&log$=prottop&blast_rank=1&RID=9WWKB7NX014) | ENSP00000352510\|TECPR2 |
| vps37a | 1 | 387 | 99 | 63 | 3 | 2.00E-167 | [AAH59584.1](http://www.ncbi.nlm.nih.gov/protein/37590372?report=genbank&log$=prottop&blast_rank=1&RID=9WWKB7NX014) | ENSP00000318629\|VPS37A |
| zfyve26 | 13 | 2552 | 98 | 43 | 4 | 0.00E+00 | [AHH02828.1](http://www.ncbi.nlm.nih.gov/protein/576010105?report=genbank&log$=prottop&blast_rank=1&RID=9WWKB7NX014) | ENSP00000450603\|ZFYVE26 |
| zfyve27 | 1 | 409 | 97 | 49 | 5 | 9.00E-119 | [NP_001070759.2](http://www.ncbi.nlm.nih.gov/protein/217272855?report=genbank&log$=prottop&blast_rank=1&RID=9V8WZT5K015) | ENSP00000353069\|ZFYVE27 |
|  | Md | 551 |  |  |  |  |  |  |
|  | SEM | 105 |  |  |  |  |  |  |

**Table 6. Intellectual Disability Gene Orthologues Blast results. Ch., chromosome; aa, protein length in amino acids; Iso., isoforms (ENSMBL.org/NCBI.gov).**

| **Gene** | **Ch** | **aa** | **Query Cover %** | **% Protein ID** | **Iso.** | **E-Value** | **Zebrafish Protein NCBI/ENSEMBL ID** | **Human Protein NCBI/ENSEMBL ID** |
| --- | --- | --- | --- | --- | --- | --- | --- | --- |
| arhgef9a | 5 | 521 | 99 | 83 | 3 | 0 | [XP_001923441.1](http://www.ncbi.nlm.nih.gov/protein/189518885?report=genbank&log$=prottop&blast_rank=5&RID=9DDADSUK013) | ENSP00000253401\|ARHGEF9 |
| arhgef9b | 14 | 1083 | 99 | 84 | 8 | 0 | [XP_009306009.1](http://www.ncbi.nlm.nih.gov/protein/688584624?report=genbank&log$=prottop&blast_rank=1&RID=9DDADSUK013) | ENSP00000253401\|ARHGEF9 |
| atp7a | 14 | 1501 | 82 | 60.7 | 2 | 0 | [NM_001042720](http://www.ncbi.nlm.nih.gov/nuccore/NM_001042720) | ENSP00000343026\|ATP7A |
| atrx | 14 | 2013 | 65 | 67 | 5 | 0 | [NP_956947.2](http://www.ncbi.nlm.nih.gov/protein/319655732?report=genbank&log$=prottop&blast_rank=1&RID=EKFC4KEC014) | ENSP00000362441\|ATRX |
| atrxl | 5 | 1764 | 58 | 49 | 4 | 0 | [XP_009299689.1](http://www.ncbi.nlm.nih.gov/protein/688554593?report=genbank&log$=prottop&blast_rank=2&RID=EKFC4KEC014) | ENSP00000362441\|ATRX |
| bcor | 22 | 1796 | 70 | 48 | 2 | 0 | [XP_005174000.1](http://www.ncbi.nlm.nih.gov/protein/528515562?report=genbank&log$=prottop&blast_rank=1&RID=EKFC4KEC014) | ENSP00000345923\|BCOR |
| caska | 9 | 920 | 99 | 93 | 7 | 0 | [NP_694420.1](http://www.ncbi.nlm.nih.gov/protein/23308741?report=genbank&log$=prottop&blast_rank=2&RID=EKFC4KEC014) | ENSP00000367408\|CASK |
| caskb | 6 | 921 | 99 | 94 | 2 | 0 | [NP_001135848.1](http://www.ncbi.nlm.nih.gov/protein/215422311?report=genbank&log$=prottop&blast_rank=1&RID=EKFC4KEC014) | ENSP00000367408\|CASK |
| cdkl5 | 11 | 1039 | 85 | 59 | 3 | 0 | [XP_005169430.1](http://www.ncbi.nlm.nih.gov/protein/528494556?report=genbank&log$=prottop&blast_rank=1&RID=EKFC4KEC014) | ENSP00000369325\|CDKL5 |
| cul4b | 14 | 864 | 95 | 85 | 2 | 0 | [NP_001116316.1](http://www.ncbi.nlm.nih.gov/protein/170932540?report=genbank&log$=prottop&blast_rank=1&RID=9DDADSUK013) | ENSP00000338919\|CUL4B |
| dmd | 1 | 3609 | 99 | 58 | 10 | 0 | [XP_009304312.1](http://www.ncbi.nlm.nih.gov/protein/688531359?report=genbank&log$=prottop&blast_rank=1&RID=EKFC4KEC014) | ENSP00000354923\|DMD |
| fancb | 9 | 807 | 98 | 25 | 1 | 4.00E-70 | [NP_001035726.1](http://www.ncbi.nlm.nih.gov/protein/95147329?report=genbank&log$=prottop&blast_rank=1&RID=EKFC4KEC014) | ENSP00000326819\|FANCB |
| gk | 1 | 563 | 99 | 79 | 7 | 0 | [XP_005159997.1](http://www.ncbi.nlm.nih.gov/protein/528468241?report=genbank&log$=prottop&blast_rank=1&RID=EKFC4KEC014) | ENSP00000368226\|GK |
| hdac8 | 7 | 378 | 99 | 73 | 2 | 0 | [NP_998596.1](http://www.ncbi.nlm.nih.gov/protein/47087397?report=genbank&log$=prottop&blast_rank=1&RID=EKFC4KEC014) | ENSP00000362674\|HDAC8 |
| kdm5c | 8 | 1559 | 94 | 63 | 2 | 0 | [XP_009302409.1](http://www.ncbi.nlm.nih.gov/protein/688568681?report=genbank&log$=prottop&blast_rank=1&RID=EKFC4KEC014) | ENSP00000364528\|KDM5C |
| mid1 | 9 | 667 | 99 | 80 | 2 | 0 | [XP_002663478.1](http://www.ncbi.nlm.nih.gov/protein/292617847?report=genbank&log$=prottop&blast_rank=1&RID=9DDADSUK013) | ENSP00000312678\|MID1 |
| ndp | 9 | 188 | 85 | 69 | 1 | 4.00E-56 | [XP_009303083.1](http://www.ncbi.nlm.nih.gov/protein/688571797?report=genbank&log$=prottop&blast_rank=1&RID=EKFC4KEC014) | ENSP00000367301\|NDP |
| nhsa | 23 | 1509 | 81 | 57 | 3 | 0 | [XP_009304395.1](http://www.ncbi.nlm.nih.gov/protein/688577615?report=genbank&log$=prottop&blast_rank=1&RID=EKFC4KEC014) | ENSP00000369400\|NHS |
| nhsb | 11 | 1609 | 79 | 55 | 3 | 0 | [XP_009295377.1](http://www.ncbi.nlm.nih.gov/protein/688612686?report=genbank&log$=prottop&blast_rank=4&RID=EKFC4KEC014) | ENSP00000369400\|NHS |
| ofd1 | 9 | 954 | 71 | 42 | 4 | 2.00E-150 | [XP_009303289.1](http://www.ncbi.nlm.nih.gov/protein/688572723?report=genbank&log$=prottop&blast_rank=1&RID=EKFC4KEC014) | ENSP00000344314\|OFD1 |
| pcdh19 | 14 | 1088 | 99 | 70 | 7 | 0.00E+00 | [NP_001120991.2](http://www.ncbi.nlm.nih.gov/protein/237681077?report=genbank&log$=prottop&blast_rank=1&RID=9DDADSUK013) | ENSP00000255531\|PCDH19 |
| pdha1a | 5 | 400 | 99 | 74 | 3 | 0 | [XP_005165191.1](http://www.ncbi.nlm.nih.gov/protein/528478848?report=genbank&log$=prottop&blast_rank=3&RID=EKFC4KEC014) | ENSP00000369134\|PDHA1 |
| pdha1b | 24 | 400 | 86 | 81 | 3 | 0 | [XP_005162743.1](http://www.ncbi.nlm.nih.gov/protein/528519711?report=genbank&log$=prottop&blast_rank=1&RID=EKFC4KEC014) | ENSP00000369134\|PDHA1 |
| pgk1 | 21 | 417 | 99 | 88 | 1 | 0 | [NP_998552.1](http://www.ncbi.nlm.nih.gov/protein/47087077?report=genbank&log$=prottop&blast_rank=1&RID=EKFC4KEC014) | ENSP00000362413\|PGK1 |
| pqbp1 | 8 | 261 | 46 | 60 | 2 | 1.00E-35 | [NP_001002435.1](http://www.ncbi.nlm.nih.gov/protein/50539938?report=genbank&log$=prottop&blast_rank=1&RID=EKFC4KEC014) | ENSP00000218224\|PQBP1 |
| ptchd1 | 24 | 887 | 99 | 71 | 2 | 0 | [XP_690754.1](http://www.ncbi.nlm.nih.gov/protein/68404314?report=genbank&log$=prottop&blast_rank=1&RID=EKFC4KEC014) | ENSP00000368666\|PTCHD1 |
| rps6ka3 | 24 | 739 | 99 | 86 | 5 | 0 | [XP_009295647.1](http://www.ncbi.nlm.nih.gov/protein/688614026?report=genbank&log$=prottop&blast_rank=1&RID=EKFC4KEC014) | ENSP00000368884\|RPS6KA3 |
| rps6ka3l | 14 | 739 | 99 | 82 | 1 | 0 | [NP_001076495.2](http://www.ncbi.nlm.nih.gov/protein/849527425?report=genbank&log$=prottop&blast_rank=8&RID=EKFC4KEC014) | ENSP00000368884\|RPS6KA3 |
| smc1a | 23 | 1233 | 99 | 90 | 2 | 0 | [NP_997975.2](http://www.ncbi.nlm.nih.gov/protein/339895751?report=genbank&log$=prottop&blast_rank=1&RID=EKFC4KEC014) | ENSP00000323421\|SMC1A |
| syn1 | 8 | 670 | 99 | 59 | 2 | 0 | [NP_001119909.1](http://www.ncbi.nlm.nih.gov/protein/187607906?report=genbank&log$=prottop&blast_rank=1&RID=9DDADSUK013) | ENSP00000295987\|SYN1 |
| ube2a | 14 | 152 | 99 | 98 | 2 | 1.00E-106 | [NP_958430.1](http://www.ncbi.nlm.nih.gov/protein/41152052?report=genbank&log$=prottop&blast_rank=1&RID=9DDADSUK013) | ENSP00000335027\|UBE2A |
| upf3a | 9 | 452 | 98 | 53 | 5.00E+00 | 1.00E-102 | [XP_005167908.1](http://www.ncbi.nlm.nih.gov/protein/528490568?report=genbank&log$=prottop&blast_rank=1&RID=9DDADSUK013) | ENSP00000364448\|UPF3A |
| upf3b | 14 | 467 | 99 | 55 | 3.00E+00 | 2.00E-105 | [NP_957248.1](http://www.ncbi.nlm.nih.gov/protein/41055672?report=genbank&log$=prottop&blast_rank=1&RID=9DDADSUK013) | ENSP00000276201\|UPF3B |
|  | Md | 875.5 |  |  |  |  |  |  |
|  | SEM | 136 |  |  |  |  |  |  |

**Table 7. Schizophrenia Gene Orthologues Blast results. Ch., chromosome; aa, protein length in amino acids; Iso., isoforms (ENSMBL.org/NCBI.gov).**

| **Gene** | **Ch** | **aa** | **Query Cover %** | **% Protein ID** | **Iso.** | **E-Value** | **Zebrafish Protein NCBI/ENSEMBL ID** | **Human Protein NCBI/ENSEMBL ID** |
| --- | --- | --- | --- | --- | --- | --- | --- | --- |
| bdnf | 7 | 278 | 99 | 70 | 5 | 1.00E-124 | [NP_001295577.1](http://www.ncbi.nlm.nih.gov/protein/821324795?report=genbank&log$=prottop&blast_rank=1&RID=7JWPX60D014) | ENSP00000320002\|BDNF |
| cacna1Ia | 3 | 2104 | 89 | 65 | 2 | 0 | [XP_009297749.1](http://www.ncbi.nlm.nih.gov/protein/688543085?report=genbank&log$=prottop&blast_rank=1&RID=7K1ENDP3014) | ENSP00000384093\|CACNA1I |
| cacna1ib | 6 | 1997 | / | 65 | 2 | 0 | ENSDARP00000139492.1 | ENSP00000384093\|CACNA1I |
| cacnb2a | 7 | 636 |  | 88 | 9 | 0 | F1QX81 (uniprot) | ENSP00000320025\|CACNB2 |
| cacnb2b | 2 | 377 |  |  |  |  |  | ENSP00000320025\|CACNB2 |
| csmd1a | 13 | 1872 | 99 | 71 | 1 | 0 | [XP_009305370.1](http://www.ncbi.nlm.nih.gov/protein/688581884?report=genbank&log$=prottop&blast_rank=4&RID=7K1ENDP3014) | ENSP00000430733\|CSMD1 |
| disc1 | 13 | 944 | 60 | 37 | 4 | 2.00E-76 | [NP_001135735.1](http://www.ncbi.nlm.nih.gov/protein/214010133?report=genbank&log$=prottop&blast_rank=1&RID=7JWPX60D014) | ENSP00000403888\|DISC1 |
| grin2aa | 3 | 1460 | 98 | 68 | 2 | 0 | [XP_691754.3](http://www.ncbi.nlm.nih.gov/protein/528474133?report=genbank&log$=prottop&blast_rank=1&RID=7JWPX60D014) | ENSP00000332549\|GRIN2A |
| grin2ab | 1 | 1445 | 99 | 55 | 2 | 0 | [XP_009304490.1](http://www.ncbi.nlm.nih.gov/protein/688531473?report=genbank&log$=prottop&blast_rank=2&RID=7JWPX60D014) | ENSP00000332549\|GRIN2A |
| grm3 | 18 | 884 | 96 | 78 | 2 | 0 | [NP_001121815.1](http://www.ncbi.nlm.nih.gov/protein/190358558?report=genbank&log$=prottop&blast_rank=1&RID=7JWPX60D014) | ENSP00000355316\|GRM3 |
| mmp16b | 2 | 613 | 99 | 72 | 2 | 0 | XP_700781.6 | ENSP00000286614\|MMP16 |
| srr | 4 | 323 | 82 | 32 | 1 | 2.00E-24 | XP_002661511.2 | ENSP00000339435\|SRR |
|  | Md | 914 |  |  |  |  |  |  |
|  | SEM | 180 |  |  |  |  |  |  |

**Table 8. Gene Ontology enrichment analysis for all ASD gene orthologues (n=35)**

| GO biological process complete | Homo sapiens - REFLIST (20814) | upload_1 (27) | upload_1 (expected) | upload_1 (over/under) | upload_1 (fold Enrichment) | upload_1 (P-value) |
| --- | --- | --- | --- | --- | --- | --- |
| positive regulation of excitatory postsynaptic potential (GO:2000463) | 19 | 3 | 0.02 | + | > 100 | 1.70E-02 |
| modulation of excitatory postsynaptic potential (GO:0098815) | 26 | 3 | 0.03 | + | 88.95 | 4.33E-02 |
| startle response (GO:0001964) | 27 | 3 | 0.04 | + | 85.65 | 4.85E-02 |
| multicellular organismal response to stress (GO:0033555) | 73 | 4 | 0.09 | + | 42.24 | 1.94E-02 |
| learning (GO:0007612) | 124 | 6 | 0.16 | + | 37.3 | 9.24E-05 |
| multi-organism behavior (GO:0051705) | 83 | 4 | 0.11 | + | 37.15 | 3.21E-02 |
| dendrite development (GO:0016358) | 85 | 4 | 0.11 | + | 36.28 | 3.52E-02 |
| regulation of synaptic plasticity (GO:0048167) | 133 | 5 | 0.17 | + | 28.98 | 5.95E-03 |
| learning or memory (GO:0007611) | 213 | 7 | 0.28 | + | 25.33 | 6.78E-05 |
| cognition (GO:0050890) | 245 | 8 | 0.32 | + | 25.17 | 5.21E-06 |
| regulation of synapse structure or activity (GO:0050803) | 218 | 6 | 0.28 | + | 21.22 | 2.52E-03 |
| modulation of synaptic transmission (GO:0050804) | 266 | 6 | 0.35 | + | 17.39 | 7.96E-03 |
| single-organism behavior (GO:0044708) | 392 | 8 | 0.51 | + | 15.73 | 1.98E-04 |
| behavior (GO:0007610) | 527 | 9 | 0.68 | + | 13.17 | 1.03E-04 |
| cell morphogenesis involved in neuron differentiation (GO:0048667) | 697 | 11 | 0.9 | + | 12.17 | 3.66E-06 |
| neuron projection morphogenesis (GO:0048812) | 714 | 11 | 0.93 | + | 11.88 | 4.71E-06 |
| neuron projection development (GO:0031175) | 838 | 12 | 1.09 | + | 11.04 | 1.39E-06 |
| axon guidance (GO:0007411) | 560 | 8 | 0.73 | + | 11.01 | 3.00E-03 |
| neuron projection guidance (GO:0097485) | 560 | 8 | 0.73 | + | 11.01 | 3.00E-03 |
| axonogenesis (GO:0007409) | 651 | 9 | 0.84 | + | 10.66 | 6.25E-04 |
| axon development (GO:0061564) | 675 | 9 | 0.88 | + | 10.28 | 8.50E-04 |
| cell morphogenesis involved in differentiation (GO:0000904) | 830 | 11 | 1.08 | + | 10.22 | 2.27E-05 |
| neuron development (GO:0048666) | 982 | 13 | 1.27 | + | 10.21 | 4.79E-07 |
| cell projection morphogenesis (GO:0048858) | 915 | 11 | 1.19 | + | 9.27 | 6.23E-05 |
| cell part morphogenesis (GO:0032990) | 935 | 11 | 1.21 | + | 9.07 | 7.79E-05 |
| head development (GO:0060322) | 699 | 8 | 0.91 | + | 8.82 | 1.57E-02 |
| neuron differentiation (GO:0030182) | 1168 | 13 | 1.52 | + | 8.58 | 4.04E-06 |
| chemotaxis (GO:0006935) | 779 | 8 | 1.01 | + | 7.92 | 3.50E-02 |
| taxis (GO:0042330) | 779 | 8 | 1.01 | + | 7.92 | 3.50E-02 |
| cell projection organization (GO:0030030) | 1172 | 12 | 1.52 | + | 7.89 | 6.18E-05 |
| cell morphogenesis (GO:0000902) | 1111 | 11 | 1.44 | + | 7.63 | 4.56E-04 |
| cellular component morphogenesis (GO:0032989) | 1191 | 11 | 1.54 | + | 7.12 | 9.24E-04 |
| generation of neurons (GO:0048699) | 1551 | 14 | 2.01 | + | 6.96 | 9.97E-06 |
| neurogenesis (GO:0022008) | 1628 | 14 | 2.11 | + | 6.63 | 1.87E-05 |
| cell development (GO:0048468) | 1776 | 14 | 2.3 | + | 6.08 | 5.77E-05 |
| nervous system development (GO:0007399) | 2271 | 16 | 2.95 | + | 5.43 | 1.25E-05 |
| system process (GO:0003008) | 1773 | 11 | 2.3 | + | 4.78 | 4.76E-02 |
| organ development (GO:0048513) | 2784 | 17 | 3.61 | + | 4.71 | 2.39E-05 |
| anatomical structure morphogenesis (GO:0009653) | 2341 | 13 | 3.04 | + | 4.28 | 1.54E-02 |
| cell differentiation (GO:0030154) | 3372 | 16 | 4.37 | + | 3.66 | 3.72E-03 |
| system development (GO:0048731) | 4011 | 19 | 5.2 | + | 3.65 | 8.90E-05 |
| cellular developmental process (GO:0048869) | 3543 | 16 | 4.6 | + | 3.48 | 7.44E-03 |
| multicellular organismal development (GO:0007275) | 4571 | 19 | 5.93 | + | 3.2 | 8.28E-04 |
| anatomical structure development (GO:0048856) | 4699 | 19 | 6.1 | + | 3.12 | 1.32E-03 |
| cellular component organization (GO:0016043) | 5066 | 19 | 6.57 | + | 2.89 | 4.64E-03 |
| cellular component organization or biogenesis (GO:0071840) | 5188 | 19 | 6.73 | + | 2.82 | 6.88E-03 |
| single-organism developmental process (GO:0044767) | 5209 | 19 | 6.76 | + | 2.81 | 7.36E-03 |
| developmental process (GO:0032502) | 5291 | 19 | 6.86 | + | 2.77 | 9.52E-03 |
| single-multicellular organism process (GO:0044707) | 6193 | 20 | 8.03 | + | 2.49 | 2.00E-02 |
| multicellular organismal process (GO:0032501) | 6444 | 20 | 8.36 | + | 2.39 | 3.95E-02 |

**Table 9. Gene Ontology enrichment analysis for duplicated ASD gene orthologues (n=17)**

| GO biological process complete | Homo sapiens - REFLIST (20814) | upload_1 (15) | upload_1 (expected) | upload_1 (over/under) | upload_1 (fold Enrichment) | upload_1 (P-value) |
| --- | --- | --- | --- | --- | --- | --- |
| startle response (GO:0001964) | 27 | 3 | 0.02 | + | > 100 | 7.63E-03 |
| dendritic spine development (GO:0060996) | 15 | 3 | 0.01 | + | > 100 | 1.32E-03 |
| postsynaptic density assembly (GO:0097107) | 5 | 2 | 0 | + | > 100 | 4.70E-02 |
| postsynaptic density organization (GO:0097106) | 5 | 2 | 0 | + | > 100 | 4.70E-02 |
| intraspecies interaction between organisms (GO:0051703) | 46 | 3 | 0.03 | + | 90.5 | 3.74E-02 |
| social behavior (GO:0035176) | 46 | 3 | 0.03 | + | 90.5 | 3.74E-02 |
| dendrite morphogenesis (GO:0048813) | 48 | 3 | 0.03 | + | 86.72 | 4.25E-02 |
| multi-organism behavior (GO:0051705) | 83 | 4 | 0.06 | + | 66.87 | 2.59E-03 |
| dendrite development (GO:0016358) | 85 | 4 | 0.06 | + | 65.3 | 2.85E-03 |
| learning (GO:0007612) | 124 | 4 | 0.09 | + | 44.76 | 1.27E-02 |
| synapse organization (GO:0050808) | 136 | 4 | 0.1 | + | 40.81 | 1.83E-02 |
| learning or memory (GO:0007611) | 213 | 5 | 0.15 | + | 32.57 | 2.41E-03 |
| cognition (GO:0050890) | 245 | 5 | 0.18 | + | 28.32 | 4.78E-03 |
| cell morphogenesis involved in neuron differentiation (GO:0048667) | 697 | 9 | 0.5 | + | 17.92 | 1.72E-06 |
| single-organism behavior (GO:0044708) | 392 | 5 | 0.28 | + | 17.7 | 4.73E-02 |
| neuron projection morphogenesis (GO:0048812) | 714 | 9 | 0.51 | + | 17.49 | 2.12E-06 |
| neuron projection development (GO:0031175) | 838 | 10 | 0.6 | + | 16.56 | 2.17E-07 |
| behavior (GO:0007610) | 527 | 6 | 0.38 | + | 15.8 | 8.42E-03 |
| cell morphogenesis involved in differentiation (GO:0000904) | 830 | 9 | 0.6 | + | 15.05 | 7.97E-06 |
| axonogenesis (GO:0007409) | 651 | 7 | 0.47 | + | 14.92 | 1.17E-03 |
| axon development (GO:0061564) | 675 | 7 | 0.49 | + | 14.39 | 1.50E-03 |
| neuron development (GO:0048666) | 982 | 10 | 0.71 | + | 14.13 | 1.03E-06 |
| cell projection morphogenesis (GO:0048858) | 915 | 9 | 0.66 | + | 13.65 | 1.87E-05 |
| cell part morphogenesis (GO:0032990) | 935 | 9 | 0.67 | + | 13.36 | 2.26E-05 |
| neuron differentiation (GO:0030182) | 1168 | 10 | 0.84 | + | 11.88 | 5.57E-06 |
| cell projection organization (GO:0030030) | 1172 | 10 | 0.84 | + | 11.84 | 5.75E-06 |
| cell morphogenesis (GO:0000902) | 1111 | 9 | 0.8 | + | 11.24 | 1.02E-04 |
| cellular component morphogenesis (GO:0032989) | 1191 | 9 | 0.86 | + | 10.49 | 1.86E-04 |
| generation of neurons (GO:0048699) | 1551 | 10 | 1.12 | + | 8.95 | 8.68E-05 |
| neurogenesis (GO:0022008) | 1628 | 10 | 1.17 | + | 8.52 | 1.38E-04 |
| cell development (GO:0048468) | 1776 | 10 | 1.28 | + | 7.81 | 3.19E-04 |
| nervous system development (GO:0007399) | 2271 | 10 | 1.64 | + | 6.11 | 3.32E-03 |

**Table 10. Gene Ontology enrichment analysis for duplicated ASD-ID gene orthologues (n=12)**

| GO biological process complete | Homo sapiens - REFLIST (20814) | upload_1 (11) | upload_1 (expected) | upload_1 (over/under) | upload_1 (fold Enrichment) | upload_1 (P-value) |
| --- | --- | --- | --- | --- | --- | --- |
| regulation of synaptic plasticity (GO:0048167) | 133 | 4 | 0.07 | + | 56.91 | 4.13E-03 |
| regulation of synapse structure or activity (GO:0050803) | 218 | 5 | 0.12 | + | 43.4 | 4.30E-04 |
| modulation of synaptic transmission (GO:0050804) | 266 | 5 | 0.14 | + | 35.57 | 1.15E-03 |
| neuron differentiation (GO:0030182) | 1168 | 7 | 0.62 | + | 11.34 | 3.68E-03 |
| generation of neurons (GO:0048699) | 1551 | 8 | 0.82 | + | 9.76 | 9.93E-04 |
| neurogenesis (GO:0022008) | 1628 | 8 | 0.86 | + | 9.3 | 1.45E-03 |
| nervous system development (GO:0007399) | 2271 | 9 | 1.2 | + | 7.5 | 7.62E-04 |

**Table 11. Gene Ontology enrichment analysis for duplicated ID gene orthologues (n=7)**

| GO biological process complete | Homo sapiens - REFLIST (20814) | upload_1 (7) | upload_1 (expected) | upload_1 (over/under) | upload_1 (fold Enrichment) | upload_1 (P-value) |
| --- | --- | --- | --- | --- | --- | --- |
| cell projection morphogenesis (GO:0048858) | 915 | 5 | 0.31 | + | 16.25 | 2.49E-02 |
| cell part morphogenesis (GO:0032990) | 935 | 5 | 0.31 | + | 15.9 | 2.77E-02 |
| neuron development (GO:0048666) | 982 | 5 | 0.33 | + | 15.14 | 3.52E-02 |

**Table 12. Gene Ontology enrichment analysis for duplicated CMT gene orthologues (n=9)**

| GO biological process complete | Homo sapiens - REFLIST (20814) | upload_1 (9) | upload_1 (expected) | upload_1 (over/under) | upload_1 (fold Enrichment) | upload_1 (P-value) |
| --- | --- | --- | --- | --- | --- | --- |
| nervous system development (GO:0007399) | 2271 | 7 | 0.98 | + | 7.13 | 4.22E-02 |

**Table 13. *in situ* hybridization data for zebrafish ASD gene orthologues.**

| Gene | Zygote  (1-64 cell) | Blastula (128 cell to 30% epiboly) | Gastrula (50% epiboly to bud) | Segment. (1-somite to 26 somite) | Pharyngula  (Prim-5 to High-pec) | Hatching (Long-pec to protruding-mouth) |
| --- | --- | --- | --- | --- | --- | --- |
| *ank2b*  (Thisse and Thisse, 2004) | - | - | - | Early:  ant-nk +  Late:  ant-fb +++  hb ++  sc ++ | Early:  fb +++  mb-TeO +++  hb+CeP +++  sc ++  Late:  br +++  sc + | - |
| *auts2* | - | - | - | - | Early:  ant-fb ++  md ++  hb ++  sc + | Early:  br +++  Late:  fb +++  md +++  cep +++  hb + |
| *cacna1ha*  (Thisse and Thisse, 2004) | - | - | - | Early:  ad ++  Late:  n.dien +++  br + | Early:  n.dien +++  br +  Late:  n.dien +++  br ++ | Early:  br ++ |
| *chd8*  (Bernier et al., 2014) | mat + | - | - | Early:  w + | Early:  br +++  sc +  Late:  br +++ | 72 hpf:  br +++  gi ++  (same for larval) |
| *cntn4*  (Gomez et al., 2012) | - | - | no expression | - | Early:  ob +++  ven-br ++  n.sc +++  vas.  cv +++  da +++  cht +++  isv +++ | - |
| *cntnap2a*  (Hoffman et al. 2016; Pujol-Marti et al., 2012) | - | - | - | - | Early:  ant-br +++  post-br  ++ | Early:  br  +++  Late:  lat. gang +++  cns + |
| *cntnap2b*  (Hoffman et al. 2016; Xing et al., 2012) | - | - | - | - | Early:  ant-tele +++  post-dien  +++  br  ++ | Early:  TeO +++  mhb  +++  hb  +++  tele  +++  br  ++  Late:  ant. fb +++  br + |
| *dscama*  (Yimlamai et al., 2005) | absent | absent  protein ubi | absent  protein ubi | Late:  n.tele ++  n.dien ++  n.mese ++ | Early:  tele ++  dien ++  mese ++  sc ++  Late:  br +++ | 5 dpf:  br +++ |
| *kdm5ba*  (Kudoh et al., 2001) | - | - | - | Early:  ant-nk +++  tb +++  ubi + | Early:  tb +++ |  |
| *kdm5bb*  (Thisse et al., 2001) |  |  |  | Early:  mhb +  rhom6 +  som +  Late:  rhom6 ++ | Early:  tele ++  ven-dien ++  teg ++  hb ++  cns ++  Late:  br +++  cns + | Early:  br +++ |
| *med13a*  (Thisse and Thisse, 2004) | - | - | - | ubi + | Early:  cns +  br ++  tb ++ | br ++  sc + |
| *ptena*  (Croushore et al., 2005) | - | - | - | Early:  ubi +++  Late:  cns +++ | Early:  sc ++  br  tele +++  hb +++  ret +++  vasc. ++ | Early:  sc ++  br +++  br.arch +++  5 dpf:  sc ++  br.arch +++  gut ++ |
| *ptenb*  (Croushore et al., 2005;Yeh et al., 2011) | maternally loaded | ubi +++ | Early:  ubi +  Late:  ubi ++ | Early:  ubi ++  Late:  som +++ | Early:  br  pos-tele +++  dien +++  teg +++ | Early:  sc ++  br +++  br.arch +++  5 dpf:  sc ++  br +++ |
| *reln*  (Anichtchik et al., 2008;Imai et al., 2012) | - | - | - | Late:  tele +++  br ++ | Early:  tele +++  ven-dien ++  Teg +++  rhom +++ | Late:  tele +++  Teg +++  rhom +++ |
| *scn1a*  (Thisse et al., 2004) | - | - | - | Early:  nt +  Late:  n.sc +++  c.gang ++  olf. pl. +++  nt ++ | Early:  c.gang +++  olf. +++  n.sc +++ | Late:  c.gang +++ |
| *scn1lab*  (Baraban et al., 2013; Novak et al., 2006) | - | - | - | - | Early:  Hb ++  sc +++  Late:  vent-hb ++  ns ++ | Early:  br +++  5 dpf:  br +++  ht ++ |
| *shank3a*  (Kozol et al., 2015) | - | - | - | - | - | Early:  fb +++  md +++  hb +++  sc ++ |
| *shank3b*  (Kozol et al., 2015) | - | - | - | - | - | Early:  fb +++  md ++  hb ++ |
| *suv420h*  (Thisse and Thisse, 2004) | - | - | ubi + | ubi +++ | br +++ sc ++ | br +++  sc + |
| *syngap1a*  (Kozol et al., 2015) | - | - | - | - | - | Early:  dor-hb +++  cep +++  teo ++  fb + |
| *syngap1b*  (Kozol et al., 2015) | - | - | - | - | - | Early:  hb +++  teo +++  dien ++ |
| *tbr1a*  (Thisse and Thisse, 2004) | - | - | - | - | olf plac +++ | olf organ +++ |
| *tbr1b*  (Thisse and Thisse, 2004) | - | - | - | tele ++ | dor-tele +++  ant-dien +++ | Early:  dor-tele +++  ant-dien +++ |

Abbreviations: anterior, ant-; posterior, pos-; dorsal, dor-; ventral, ven-; maternally loaded, mat; whole organism, w; neural keel, nk; whole brain, br; nuclei, n.; telencephalon, tele; diencephalon, dien; mesencephalon, mese; rhomoncephalon, rhom; forebrain, fb; midbrain, mb; hindbrain, hb; optic tectum, TeO; cerebellar plate, CeP; adaxial cells, ad; vasculature, vas; cranial vasculature, cv; dorsal aorta, da; intersegmental vessels, isv; caudal hematopoietic, cht; lateralis ganglia, lat. gang.; tail bud, tb; heart, ht; statoacoustic ganglia, sag; placode, plac; olfactory, olf;

**Table 14. *in situ* hybridization data for zebrafish ASD-ID gene orthologues.**

| Gene | Zygote  (1-64 cell) | Blastula (128 cell to 30% epiboly) | Gastrula (50% epiboly to bud) | Segment. (1-somite to 26 somite) | Pharyngula  (Prim-5 to High-pec) | Hatching (Long-pec to protruding-mouth) |
| --- | --- | --- | --- | --- | --- | --- |
| *arhgef9a*  (Thisse and Thisse, 2005) | - | - | - | - | Early:  br + | Early:  br ++  Late:  rgc +++  teo +++  br + |
| *arhgef9b*  (Wakayama et al., 2015) | - | - | - | - | Early:  ht +++  da +++  ven-isv +++  ca +++  cv +++  Late:  tele +++  pos-teo ++  ht +++  da +++  ven-isv +++  ca +++  cv +++ | - |
| *cdh10*  (Liu et al., 2006) | - | - | - | Early:  no expression  Late:  ant-latl +++  pos-latl ++  nc +  tb + | Early:  ven-dien ++  ant-latl +++  pos-latl ++  nc + | Late:  ant-latl +++  pos-latl ++  stripe:  tele ++  dien ++  hyp ++  ven-hb +++ |
| *chd15*  (Thisse and Thisse, 2005) | - | - | - | Late:  ad +++  som +++ | Early:  myotome ++  Late:  pect +++  n.dien +++ | Early:  pect +++  n.hyp +++  myotome + |
| *il1rapl1a*  (Yoshida and Mishina, 2008) | - | - | - | - | - | Early:  tele ++  teo ++ |
| *il1rapl1b*  (Yoshida and Mishina, 2008) | - | - | - | - | - | Early:  tele +++  teo +++ |
| *nlgn3a*  (Rissone et al., 2010) |  |  |  |  | Early:  tele +++  med.dien +++  ant.n.rhom+++ | Early:  tele +++  med.dien +++  rhom +++  Late:  dien +++  rhom +++ |
| *nlgn3b*  (Rissone et al., 2010) |  |  |  |  | Early:  tele ++  med.dien +++  n.rhom +++ | Early:  pos.tele ++  med.dien +++  vent.rhom +++  Late:  dien +++  rhom +++ |
| *nlgn4a*  (Davey et al., 2010;Rissone et al., 2010) |  |  |  |  | Early:  dor.tele +++  dien ++ | Early:  dien ++  vent.rhom +++  Late:  dien +++  mese +++  rhom +++ |
| *nlgn4b*  (Davey et al., 2010;Rissone et al., 2010) |  |  |  |  | Early:  ant.tele +++  dien ++ | Early:  tele +++  med.dien +++  ven.rhom +++ |
| *pcdh10a*  (Thisse and Thisse, 2004) | not expressed | not expressed | vent-lat-axis +++ | heart +++  opt.ves ++  n.dien +++  tele ++  otic plac ++  ncrest +++ | Early:  tele +++  ven-dien +++  opt.ves +++  ant.Teg +++  otic ves +++  ncrest +++  heart ++  vent.z ++  Late:  tele +++  ven-dien +++  lens +++  Teg +++  otic ves ++  retina ++  vent.z ++ | Early:  br +++  retina +++  5 dpf:  br +++  retina +++ |
| *pcdh10b*  (Thisse and Thisse, 2005) | - | - | axial hypo  ++ | Early:  somite +++  polster +++  Late:  somite +++  epi ++  neurod ++ | Early:  epi ++  dor-mese +++  n.tele +++  tail ++  Late:  dor-Th +++  rhom ++  phr-arch ++ | dor-Th +++  rhom ++ |
| *pcdh19*  (Emond et al., 2009) | Not expressed | Not expressed | med-ant  neur-plate  ++ | Early:  Bands  tele +++  mese ++  romb +++  Late:  ven-tele ++  ven-mese +++  TeO ++  Rhom ++ | Late:  tele +  dien ++  mese +++  Rhom +++ | Early:  tele ++  dien +  dor-Th +++  prO +++  rhom +++ |
| *stxbp1a*  (Thisse and Thisse, 2004) | Not expressed | Not expressed | Not expressed | Late:  heart primordium  ++  n.sc +++  pos-gang+++ | Early:  tele +++  Olf.plac ++  ven-mese +++  rhom +++  sc +++  Late  tele +++  Olf.plac ++  med-ret +++  mese +++  rhom +++  sc +++ | Early:  tele +++  Olf.plac ++  RGC +++  mese +++  rhom +++  sc +++  5 Dpf:  tele +  RGC +++  mese +++  rhom +++  sc + |
| *syn1*  (Garbarino et al., 2014) | - | Late:  epiblast ++ | Late:  nk ++ | - | Early:  n.rhomb ++  n.sc +++ | Late:  olf bulb +++  Teg +++  rhom  r2-6 +++  n.sc +++ |
| *sypb*  (Meyer and Smith, 2006) | - | - | - | - | - | Late:  ipl +++  lat-mese +++  n.mese +++  n.sc +++ |

**Table 15. *in situ* hybridization data for zebrafish ID gene orthologues.**

| Gene | Zygote  (1-64 cell) | Blastula (128 cell to 30% epiboly) | Gastrula (50% epiboly to bud) | Segment. (1-somite to 26 somite) | Pharyngula  (Prim-5 to High-pec) | Hatching (Long-pec to protruding-mouth) |
| --- | --- | --- | --- | --- | --- | --- |
| *fancb*  *(Titus et al., 2009)* | - | - | - | Late:  cns +++  icm +++ | Early:  Brain +++ | Early:  Not expressed |
| *atp7a*  (Mendelsohn et al., 2006) | Maternally loaded | - | - | Early:  ubi +  nc ++ | Early:  ventricle ++  nc ++ | 5 dpf:  Lens +++  mese ++  liver +++ |
| *caska*  (Thisse and Thisse, 2004) | - | - | ysl ++ | Early:  ysl +++  Late:  ysl +++  opt. ++  nc ++ | Early:  ysl +++  opt. +++  nc ++  Late:  ysl ++  mhb +++  retina +++ | Late:  retina +++  n.rhomb +++  brain ++ |
| *dmd*  (Thisse et al., 2001) | - | - | Late:  axial mesoderm+++ | Early:  nc +++  Late:  myotome+++  floor pl. ++  nc ++ | myotome +++ | myotome + |
| *nhsa*  (Mapp et al., 2011) | - | - | - | Early:  som ++  cns + | Early:  som +++  br ++ | - |
| *pdha1a*  (Thisse and Thisse, 2004) | - | - | - | Early:  ubi ++  adaxial +++  Early:  ubi ++  mese +++  myotome+++  TeO +++  proneph +++ | Early:  ubi +  heart ++  eye ++  TeO ++  Late:  brain ++  pect. +++ |  |
| *ofd1*  (Ferrante et al., 2009) | maternally loaded | - | Early:  ubi ++ | Early:  ubi +  Kup ves. ++ | Early:  latl +++ | Late:  neuromast ++ |
| *plp1a*  (Takada and Appel, 2010) | - | - | - | - | - | Late:  hb oligo +++  sc oligo +++ |
| *plp1b*  (Takada and Appel, 2010) | - | - | - | - | - | Late:  br ++  5 dpf:  n.mb/hb +++ |
| *slc16a2*  (Vatine et al., 2013) | - | - | Late:  ubi ++ | - | Early:  tele +++  mese +++  rhom +++  eye +++ | Early:  br ++  s.c. +++ |
| *smc1a*  (Monnich et al., 2009) | - | - | - | - | Early:  br + | Early:  br +  IV vent. +++  fb vent. ++  n.rhomb +++  Late:  br +  mhb ++  5 dpf:  IV vent +++  mese ++ |

**Table 16. *in situ* hybridization data for zebrafish SCZ gene orthologues.**

| Gene | Zygote  (1-64 cell) | Blastula (128 cell to 30% epiboly) | Gastrula (50% epiboly to bud) | Segment. (1-somite to 26 somite) | Pharyngula  (Prim-5 to High-pec) | Hatching (Long-pec to protruding-mouth) |
| --- | --- | --- | --- | --- | --- | --- |
| *bdnf*  (Rauch et al., 2003;Thisse and Thisse, 2004) | not expressed | not expressed | not expressed | Late:  tele +++  dien +++  n.rhom +++  n.sc +++ | Early:  tele +++  n.dien +++  Teg ++ rhom ++  latl ++  Late:  tele +++  ant-dien +++  Teg ++ rhom ++ | Late:  tele +++  n.hyp +++  Teg ++  otic +++  rhom ++  retina ++  rgl ++  5 dpf:  br +  ear +++ |
| *cacnb2a*  (Zhou et al., 2008) |  |  |  |  | Early:  trig ++  epith ++ | Early:  br ++  opt.nerve ++  epith ++  Late:  epith ++  retina +++ |
| *cacnb2b*  (Thisse and Thisse, 2004;Zhou et al., 2008) | not expressed | not expressed | not expressed | not expressed | Early:  n.rhom ++  n.sc ++ | Early:  n.rhom +++  Late:  rhom ++  mese +++  inl +++  retina ++  5 dpf:  inl +++  retina ++ |
| *csmd1*  (Thisse and Thisse, 2004) | not expressed | not expressed | not expressed | not expressed | Early:  n.ven-mese +++  n.tele ++  mese +  n.sc ++  Late:  Tele +++  n.TeO ++  n.Teg ++  n.rho +++ | br +++ |
| *disc1*  (Wood et al., 2009) | - | - | _ | Late:  ncrest +++ | Early:  ven-otic +++  ncrest +++  proneph? ++  mhb ++ | Early:  br ++  otic +++  n.crest +++  cartilage +++  pre-olig? +++ |
| *grin2aa*  (Thompson et al., 2005) | - | - | - | - | - | Early:  retina +++ |
| *grin2ab*  (Thisse and Thisse, 2004) | - | not expressed | not expressed | not expressed | not expressed | Early:  retina +++  rgl +++  mese +  rhom +  5 dpf  rgl +++  mese +  rhom + |
| *grim3*  (Haug et al., 2013) | - | - | - | - | - | Late:  dor-tele +++  periven +++  n.hyp +++  5 dpf:  dor-tele +++  n.hyp +++  cere +++  periven +++ |

**Table 17. *in situ* hybridization data for zebrafish ATX gene orthologues.**

| Gene | Zygote  (1-64 cell) | Blastula (128 cell to 30% epiboly) | Gastrula (50% epiboly to bud) | Segment. (1-somite to 26 somite) | Pharyngula  (Prim-5 to High-pec) | Hatching (Long-pec to protruding-mouth) |
| --- | --- | --- | --- | --- | --- | --- |
| *abcb7*  (Thisse et al., 2001) | - | - | not expressed | ubi ++  hb +++ | Early:  liver +++  br ++ | liver +++  br ++ |
| *atm*  (Imamura and Kishi, 2005) | maternally loaded | animal pole +++ | 50% epi  +++  Late:  Not expressed | Early:  Not expressed  Late:  ubi ++ | Early:  ubi ++  br +++  pos-tail +++ | Early:  pos-tail +++ |
| *atxn7*  (Yanicostas et al., 2012) | maternally loaded | ubi +++ | ubi +++ | br +++ | br +++ | Early:  br ++  5 dpf:  br ++  tele +++  teo +++  cer +++ |
| *cacna1aa*  (Thisse and Thisse, 2005) | - | - | - | Late:  ant-tele +++  n.dien +++  hb +++  n.sc +++ | Late:  ant-tele +++  mese +++  hb +++  n.sc ++ | Late:  tele ++  mese ++  vent-hb +++  rgc ++  sc +  5dpf:  br +++  rgc +++ |
| *cacna1ab*  (Low et al. 2012, J. neuro.) | - | - | - | - | - | Early:  rbc ++ |
| *elovl4a*  (Thisse and Thisse, 2004) | - | - | - | n.fb ++  trig.plac +++  pos-hb +++  n.sc ++ | Early:  dor-tele +++  ven-tele +++  hb +++  cep ++  sc ++  Late:  tele +++  dien +++  ven-mb +++  hb +++  ant-cep +++  sc ++ | Early:  br +++ |
| *elovl4b*  (Thisse and Thisse, 2004) | - | - | - | retina +++ | Early:  lens +++  epi +++  Late:  lens +++  opt.n +++ epi +++ | Early:  retina +++  pl  epi +++  Early:  retina +++  pl  epi +++ |
| *grid2*  (Katsuyama et al., 2007) | - | - | - | - | - | Early & Late:  pos-tele +++  teo +++  cep +++  ant-hb +++  5 dpf:  dor-cere +++ |
| *kcnc3a*  (Hsieh et al., 2014) | - | - | - | - | - | 4-6 dpf:  pcl (cere)+++ |
| *kcnd3*  (Thisse and Thisse, 2004) | - | - | - | - | Early:  ant-tele ++  pos-tele +++  n.vent-rhom  +++  n.sc + | Early:  tele ++  olfb ++  ven-teo +++  vent-hb +++  RGL ++ |
| *nop56*  (Recher et al., 2013) | - | - | - | - | - | Early:  mb  mcl ++ |
| *pdyn*  (Appelbaum et al., 2010) | - | - | - | - | - | Early:  lat hyp +++ |
| *phyh*  (Thisse and Thisse, 2004) | not expressed | not expressed | not expressed | not expressed | not expressed | Liver +++  5 dpf liver +++  intestine +++ |
| *prkcg*  (Patten et al., 2007;Patten and Ali, 2009) | - | - | - | - | - | 5-7 dpf:  mauthner ab |
| wfs1a  (Thisse and Thisse, 2004) | - | - | - | n.myotome + | Late:  br +  myotome +  cep +++ | Early:  br +  myotome +  cep +++ |

**Table 18. *in situ* hybridization data for zebrafish HSP gene orthologues.**

| Gene | Zygote  (1-64 cell) | Blastula (128 cell to 30% epiboly) | Gastrula (50% epiboly to bud) | Segment. (1-somite to 26 somite) | Pharyngula  (Prim-5 to High-pec) | Hatching (Long-pec to protruding-mouth) |
| --- | --- | --- | --- | --- | --- | --- |
| *atl1*  (Fassier et al., 2010) | - | - | - | Late:  tele +++  ven-dien +++  ven-mese +++  ven-rhom+++  sc +++ | Late:  tele +++  dien +++  ven-mese +++  rhom +++  sc +++ | - |
| *erlin2*  (Thisse and Thisse, 2004) | - | - | - | Early:  br ++ | br +++  retina +++ | br ++  retina ++  5-6 dpf:  br ++  retina ++ |
| *fa2h*  (Thisse et al., 2001) | - | - | early:  n.evl +++ | early:  evl +  late:  olf.plac +++  proneph +++ | olf-ep +++  proneph ++ | early:  olf bulb +++  pharm +++  proneph ++  gut ++ |
| *gad1b*  (Thisse et al., 2001) | - | - | - | - | early:  tele +++  dien +++  ven-mese ++  n.rhom +++  n.sc +++  late:  tele +++  dien +++  Teg +++  n.rhom +++  ven-sc ++ | early:  tele ++  dien +++  TeO +++  rhom ++  cere +++ |
| *hspd1*  (Rauch et al. 2003, zfin.org; Thisse et al. 2001, zfin.org) | maternally loaded | ubi ++ | ubi ++ | Early:  mese nk ++  opt ves ++  otic prim ++  ysl ++  adaxial +++  Late:  mese +++  venmeso ++  eye +++  som +++ | Early:  TeO +++  epi +  eye +++  cere ++  bran-arc ++  som +++  proneph ++  gut ++ | - |
| *kif5aa*  (Thisse and Thisse, 2004) | - | not expressed | not expressed | Late:  tele +  nt +  trig-plac +++  n.sc +++ | Early:  tele +++  dien +++  Teg +++  n.rhom +++  sc ++  Late:  tele +++  olf ++  dien +++  mese +++  rhom +++  cere ++  sc ++ | Early:  tele +++  olf +++  dien +++  mese +++  rhom +++  cere +++  retina +++  sc +  5 dpf:  tele +++  olf ++  dien +++  mese +++  rhom +++  cere +++  retina  gcl +++  inl +++  sc + |
| *kif5ab*  (Campbell and Marlow, 2013) | - | - | - | - | tele +  dien +  mese +  rhom +  sc + | Early:  tele ++  dien ++  mese ++  rhom ++  sc ++  4 dpf:  tele +++  dien +++  mese +++  rhom +++  sc +++ |
| *rtn2a*  (Housley et al., 2014) | - | - | - | - | sk-muscl+++  eye +  br + | - |
| *rtn2b*  (Housley et al., 2014) | - | - | - | - | sk-muscl ++  eye ++  br ++ | - |

**Table 19. *in situ* hybridization data for zebrafish CMT gene orthologues.**

| Gene | Zygote  (1-64 cell) | Blastula (128 cell to 30% epiboly) | Gastrula (50% epiboly to bud) | Segment. (1-somite to 26 somite) | Pharyngula  (Prim-5 to High-pec) | Hatching (Long-pec to protruding-mouth) |
| --- | --- | --- | --- | --- | --- | --- |
| *fgd4a*  (Thisse and Thisse, 2004) | - | - | - | - | early:  vent.dien +++  vent.teg +++  late:  br ++ | - |
| *gars*  (Thisse and Thisse, 2004) |  |  |  | late:  hb n.plate +++  dien ++  fb. nk. ++  mb.nk ++ | early:  opt. ves ++  mese ++  late:  retina ++  mb +++ | early:  mb ++  retina + |
| *gdap1*  (Thisse and Thisse, 2004) |  |  |  | late:  n.tele ++  n.dien ++ | early:  tele ++  dien ++  mese ++  rhom ++  late:  tele +++  dien +++  mese +++  rhom +++ |  |
| *mpz*  (Thisse and Thisse, 2004) | - | - | - | mb ++  rhomb +++  ant.s.c. +++ | early:  teg ++  mb ++  rhomb +++  ant. s.c. +++  lens +++  late:  teg ++  mb ++  rhomb +++  ant.s.c. +++ | early:  rhomb +++  ant.s.c. +++  5 dpf:  n.rhomb +++ |
| *wnk1b*  (Croushore et al., 2005) | - | - | - | early:  mb.n.k. ++  somite ++  late:  n.c. ++ | ubi + | ubi + |

Abbreviations: ant-, anterior; pos-, posterior; dor-, dorsal; ven-, ventra; neural keel, nk; br ,whole brain; n, nuclei; tele, telencephalon; dien, diencephalon; mese, mesencephalon; rhom, rhomoncephalon; fb, forebrain; mb, midbrain; hb, hindbrain; TeO, optic tectum; CeP, cerebellar plate; ad, adaxial cells; vas, vasculature; cv, cranial vasculature; da, dorsal aorta; isv, intersegmental vessles; cht, caudal hemtopoietic; lat, gagn., lateralis; tb, tail bud; ht, heart; sag, statoacoustic ganglia.

**IN SITU REFERENCES**

Anichtchik, O., Diekmann, H., Fleming, A., Roach, A., Goldsmith, P., and Rubinsztein, D.C. (2008). Loss of PINK1 function affects development and results in neurodegeneration in zebrafish. *J Neurosci* 28**,** 8199-8207. doi: 10.1523/JNEUROSCI.0979-08.2008.

Appelbaum, L., Wang, G., Yokogawa, T., Skariah, G.M., Smith, S.J., Mourrain, P., and Mignot, E. (2010). Circadian and homeostatic regulation of structural synaptic plasticity in hypocretin neurons. *Neuron* 68**,** 87-98. doi: 10.1016/j.neuron.2010.09.006.

Baraban, S.C., Dinday, M.T., and Hortopan, G.A. (2013). Drug screening in Scn1a zebrafish mutant identifies clemizole as a potential Dravet syndrome treatment. *Nat Commun* 4**,** 2410. doi: 10.1038/ncomms3410.

Bernier, R., Golzio, C., Xiong, B., Stessman, H.A., Coe, B.P., Penn, O., Witherspoon, K., Gerdts, J., Baker, C., Vulto-van Silfhout, A.T., Schuurs-Hoeijmakers, J.H., Fichera, M., Bosco, P., Buono, S., Alberti, A., Failla, P., Peeters, H., Steyaert, J., Vissers, L.E., Francescatto, L., Mefford, H.C., Rosenfeld, J.A., Bakken, T., O'Roak, B.J., Pawlus, M., Moon, R., Shendure, J., Amaral, D.G., Lein, E., Rankin, J., Romano, C., de Vries, B.B., Katsanis, N., and Eichler, E.E. (2014). Disruptive CHD8 mutations define a subtype of autism early in development. *Cell* 158**,** 263-276. doi: 10.1016/j.cell.2014.06.017.

Campbell, P.D., and Marlow, F.L. (2013). Temporal and tissue specific gene expression patterns of the zebrafish kinesin-1 heavy chain family, kif5s, during development. *Gene Expr Patterns* 13**,** 271-279. doi: 10.1016/j.gep.2013.05.002.

Croushore, J.A., Blasiole, B., Riddle, R.C., Thisse, C., Thisse, B., Canfield, V.A., Robertson, G.P., Cheng, K.C., and Levenson, R. (2005). Ptena and ptenb genes play distinct roles in zebrafish embryogenesis. *Dev Dyn* 234**,** 911-921. doi: 10.1002/dvdy.20576.

Davey, C., Tallafuss, A., and Washbourne, P. (2010). Differential expression of neuroligin genes in the nervous system of zebrafish. *Dev Dyn* 239**,** 703-714. doi: 10.1002/dvdy.22195.

Emond, M.R., Biswas, S., and Jontes, J.D. (2009). Protocadherin-19 is essential for early steps in brain morphogenesis. *Dev Biol* 334**,** 72-83. doi: 10.1016/j.ydbio.2009.07.008.

Fassier, C., Hutt, J.A., Scholpp, S., Lumsden, A., Giros, B., Nothias, F., Schneider-Maunoury, S., Houart, C., and Hazan, J. (2010). Zebrafish atlastin controls motility and spinal motor axon architecture via inhibition of the BMP pathway. *Nat Neurosci* 13**,** 1380-1387. doi: 10.1038/nn.2662.

Ferrante, M.I., Romio, L., Castro, S., Collins, J.E., Goulding, D.A., Stemple, D.L., Woolf, A.S., and Wilson, S.W. (2009). Convergent extension movements and ciliary function are mediated by ofd1, a zebrafish orthologue of the human oral-facial-digital type 1 syndrome gene. *Hum Mol Genet* 18**,** 289-303. doi: 10.1093/hmg/ddn356.

Garbarino, G., Costa, S., Pestarino, M., and Candiani, S. (2014). Differential expression of synapsin genes during early zebrafish development. *Neuroscience* 280**,** 351-367. doi: 10.1016/j.neuroscience.2014.09.015.

Gomez, G., Lee, J.H., Veldman, M.B., Lu, J., Xiao, X., and Lin, S. (2012). Identification of vascular and hematopoietic genes downstream of etsrp by deep sequencing in zebrafish. *PLoS One* 7**,** e31658. doi: 10.1371/journal.pone.0031658.

Haug, M.F., Gesemann, M., Mueller, T., and Neuhauss, S.C. (2013). Phylogeny and expression divergence of metabotropic glutamate receptor genes in the brain of zebrafish (Danio rerio). *J Comp Neurol* 521**,** 1533-1560. doi: 10.1002/cne.23240.

Housley, M.P., Reischauer, S., Dieu, M., Raes, M., Stainier, D.Y., and Vanhollebeke, B. (2014). Translational profiling through biotinylation of tagged ribosomes in zebrafish. *Development* 141**,** 3988-3993. doi: 10.1242/dev.111849.

Hsieh, J.Y., Ulrich, B., Issa, F.A., Wan, J., and Papazian, D.M. (2014). Rapid development of Purkinje cell excitability, functional cerebellar circuit, and afferent sensory input to cerebellum in zebrafish. *Front Neural Circuits* 8**,** 147. doi: 10.3389/fncir.2014.00147.

Imai, H., Oomiya, Y., Kikkawa, S., Shoji, W., Hibi, M., Terashima, T., and Katsuyama, Y. (2012). Dynamic changes in the gene expression of zebrafish Reelin receptors during embryogenesis and hatching period. *Dev Growth Differ* 54**,** 253-263. doi: 10.1111/j.1440-169X.2012.01327.x.

Imamura, S., and Kishi, S. (2005). Molecular cloning and functional characterization of zebrafish ATM. *Int J Biochem Cell Biol* 37**,** 1105-1116. doi: 10.1016/j.biocel.2004.10.015.

Katsuyama, Y., Oomiya, Y., Dekimoto, H., Motooka, E., Takano, A., Kikkawa, S., Hibi, M., and Terashima, T. (2007). Expression of zebrafish ROR alpha gene in cerebellar-like structures. *Dev Dyn* 236**,** 2694-2701. doi: 10.1002/dvdy.21275.

Kozol, R.A., Cukier, H.N., Zou, B., Mayo, V., De Rubeis, S., Cai, G., Griswold, A.J., Whitehead, P.L., Haines, J.L., Gilbert, J.R., Cuccaro, M.L., Martin, E.R., Baker, J.D., Buxbaum, J.D., Pericak-Vance, M.A., and Dallman, J.E. (2015). Two knockdown models of the autism genes SYNGAP1 and SHANK3 in zebrafish produce similar behavioral phenotypes associated with embryonic disruptions of brain morphogenesis. *Hum Mol Genet* 24**,** 4006-4023. doi: 10.1093/hmg/ddv138.

Kudoh, T., Tsang, M., Hukriede, N.A., Chen, X., Dedekian, M., Clarke, C.J., Kiang, A., Schultz, S., Epstein, J.A., Toyama, R., and Dawid, I.B. (2001). A gene expression screen in zebrafish embryogenesis. *Genome Res* 11**,** 1979-1987. doi: 10.1101/gr.209601.

Liu, Q., Duff, R.J., Liu, B., Wilson, A.L., Babb-Clendenon, S.G., Francl, J., and Marrs, J.A. (2006). Expression of cadherin10, a type II classic cadherin gene, in the nervous system of the embryonic zebrafish. *Gene Expr Patterns* 6**,** 703-710. doi: 10.1016/j.modgep.2005.12.009.

Mapp, O.M., Walsh, G.S., Moens, C.B., Tada, M., and Prince, V.E. (2011). Zebrafish Prickle1b mediates facial branchiomotor neuron migration via a farnesylation-dependent nuclear activity. *Development* 138**,** 2121-2132. doi: 10.1242/dev.060442.

Mendelsohn, B.A., Yin, C., Johnson, S.L., Wilm, T.P., Solnica-Krezel, L., and Gitlin, J.D. (2006). Atp7a determines a hierarchy of copper metabolism essential for notochord development. *Cell Metab* 4**,** 155-162. doi: 10.1016/j.cmet.2006.05.001.

Meyer, M.P., and Smith, S.J. (2006). Evidence from in vivo imaging that synaptogenesis guides the growth and branching of axonal arbors by two distinct mechanisms. *J Neurosci* 26**,** 3604-3614. doi: 10.1523/JNEUROSCI.0223-06.2006.

Monnich, M., Banks, S., Eccles, M., Dickinson, E., and Horsfield, J. (2009). Expression of cohesin and condensin genes during zebrafish development supports a non-proliferative role for cohesin. *Gene Expr Patterns* 9**,** 586-594. doi: 10.1016/j.gep.2009.08.004.

Novak, A.E., Taylor, A.D., Pineda, R.H., Lasda, E.L., Wright, M.A., and Ribera, A.B. (2006). Embryonic and larval expression of zebrafish voltage-gated sodium channel alpha subunit genes. *Dev. Dyn.* 235, 7, 1962-1973

Patten, S.A., and Ali, D.W. (2009). PKCgamma-induced trafficking of AMPA receptors in embryonic zebrafish depends on NSF and PICK1. *Proc Natl Acad Sci U S A* 106**,** 6796-6801. doi: 10.1073/pnas.0811171106.

Patten, S.A., Sihra, R.K., Dhami, K.S., Coutts, C.A., and Ali, D.W. (2007). Differential expression of PKC isoforms in developing zebrafish. *Int J Dev Neurosci* 25**,** 155-164. doi: 10.1016/j.ijdevneu.2007.02.003.

Pujol-Marti, J., Zecca, A., Baudoin, J.P., Faucherre, A., Asakawa, K., Kawakami, K., and Lopez-Schier, H. (2012). Neuronal birth order identifies a dimorphic sensorineural map. *J Neurosci* 32**,** 2976-2987. doi: 10.1523/JNEUROSCI.5157-11.2012.

Rauch, G.J., Lyons, D.A., Middendorf, I., Friedlander, B., Arana, N., Reyes, T., and Talbot, W.S. (2003). "Submission and Curation of Gene Expression Data.". (ZFIN Direct Data Submission).

Recher, G., Jouralet, J., Brombin, A., Heuze, A., Mugniery, E., Hermel, J.M., Desnoulez, S., Savy, T., Herbomel, P., Bourrat, F., Peyrieras, N., Jamen, F., and Joly, J.S. (2013). Zebrafish midbrain slow-amplifying progenitors exhibit high levels of transcripts for nucleotide and ribosome biogenesis. *Development* 140**,** 4860-4869. doi: 10.1242/dev.099010.

Rissone, A., Sangiorgio, L., Monopoli, M., Beltrame, M., Zucchi, I., Bussolino, F., Arese, M., and Cotelli, F. (2010). Characterization of the neuroligin gene family expression and evolution in zebrafish. *Dev Dyn* 239**,** 688-702. doi: 10.1002/dvdy.22196.

Takada, N., and Appel, B. (2010). Identification of genes expressed by zebrafish oligodendrocytes using a differential microarray screen. *Dev Dyn* 239**,** 2041-2047. doi: 10.1002/dvdy.22338.

Thisse, B., Pflumio, S., Furthauer, M., Loppin, B., Heyer, V., Degrave, A., Woehl, R., Lux, A., Steffan, T., Charbonnier, X.Q., and Thisse, C. (2001). "Expression of the zebrafish genome during embryogenesis.", in: *ZFIN Direct Data Submission* ).

Thisse, B., and Thisse, C. (2004). "Fast Release Clones: A High Throughput Expression Analysis. ", in: *ZFIN Direct Data Submission* ).

Thisse, C., and Thisse, B. (2005). "High Throughput Expression Analysis of ZF-Models Consortium Clones. ", in: *Zebrafish Model Organism Consortium.* (ZFIN Direct Data Submission).

Thompson, C.M., Davis, E., Carrigan, C.N., Cox, H.D., Bridges, R.J., and Gerdes, J.M. (2005). Inhibitor of the glutamate vesicular transporter (VGLUT). *Curr Med Chem* 12**,** 2041-2056.

Titus, T.A., Yan, Y.L., Wilson, C., Starks, A.M., Frohnmayer, J.D., Bremiller, R.A., Canestro, C., Rodriguez-Mari, A., He, X., and Postlethwait, J.H. (2009). The Fanconi anemia/BRCA gene network in zebrafish: embryonic expression and comparative genomics. *Mutat Res* 668**,** 117-132. doi: 10.1016/j.mrfmmm.2008.11.017.

Vatine, G.D., Zada, D., Lerer-Goldshtein, T., Tovin, A., Malkinson, G., Yaniv, K., and Appelbaum, L. (2013). Zebrafish as a model for monocarboxyl transporter 8-deficiency. *J Biol Chem* 288**,** 169-180. doi: 10.1074/jbc.M112.413831.

Wakayama, Y., Fukuhara, S., Ando, K., Matsuda, M., and Mochizuki, N. (2015). Cdc42 mediates Bmp-induced sprouting angiogenesis through Fmnl3-driven assembly of endothelial filopodia in zebrafish. *Dev Cell* 32**,** 109-122. doi: 10.1016/j.devcel.2014.11.024.

Wood, J.D., Bonath, F., Kumar, S., Ross, C.A., and Cunliffe, V.T. (2009). Disrupted-in-schizophrenia 1 and neuregulin 1 are required for the specification of oligodendrocytes and neurones in the zebrafish brain. *Hum Mol Genet* 18**,** 391-404. doi: 10.1093/hmg/ddn361.

Xing, L., Hoshijima, K., Grunwald, D.J., Fujimoto, E., Quist, T.S., Sneddon, J., Chien, C.B., Stevenson, T.J., and Bonkowsky, J.L. (2012). Zebrafish foxP2 zinc finger nuclease mutant has normal axon pathfinding. *PLoS One* 7**,** e43968. doi: 10.1371/journal.pone.0043968.

Yanicostas, C., Barbieri, E., Hibi, M., Brice, A., Stevanin, G., and Soussi-Yanicostas, N. (2012). Requirement for zebrafish ataxin-7 in differentiation of photoreceptors and cerebellar neurons. *PLoS One* 7**,** e50705. doi: 10.1371/journal.pone.0050705.

Yeh, C.M., Liu, Y.C., Chang, C.J., Lai, S.L., Hsiao, C.D., and Lee, S.J. (2011). Ptenb mediates gastrulation cell movements via Cdc42/AKT1 in zebrafish. *PLoS One* 6**,** e18702. doi: 10.1371/journal.pone.0018702.

Yimlamai, D., Konnikova, L., Moss, L.G., and Jay, D.G. (2005). The zebrafish down syndrome cell adhesion molecule is involved in cell movement during embryogenesis. *Dev Biol* 279**,** 44-57. doi: 10.1016/j.ydbio.2004.12.001.

Yoshida, T., and Mishina, M. (2008). Zebrafish orthologue of mental retardation protein IL1RAPL1 regulates presynaptic differentiation. *Mol Cell Neurosci* 39**,** 218-228. doi: 10.1016/j.mcn.2008.06.013.

Zhou, W., Horstick, E.J., Hirata, H., and Kuwada, J.Y. (2008). Identification and expression of voltage-gated calcium channel beta subunits in Zebrafish. *Dev Dyn* 237**,** 3842-3852. doi: 10.1002/dvdy.21776.
